# Enhanced Function of Induced Pluripotent Stem Cell‐Derived Endothelial Cells Through ESM1 Signaling

**DOI:** 10.1002/stem.2936

**Published:** 2018-11-17

**Authors:** Marta Vilà‐González, Sophia Kelaini, Corey Magee, Rachel Caines, David Campbell, Magdalini Eleftheriadou, Amy Cochrane, Daiana Drehmer, Marianna Tsifaki, Karla O'Neill, Edoardo Pedrini, Chunbo Yang, Reinhold Medina, Denise McDonald, David Simpson, Anna Zampetaki, Lingfang Zeng, David Grieve, Noemi Lois, Alan W. Stitt, Andriana Margariti

**Affiliations:** ^1^ Centre for Experimental Medicine Queen's University Belfast Belfast, Co Antrim United Kingdom; ^2^ Cardiovascular Division King's College London London United Kingdom

**Keywords:** ESM1, Reprogramming, Endothelial cells, Vascular disease, Induced pluripotent stem cells

## Abstract

The mortality rate for (cardio)‐vascular disease is one of the highest in the world, so a healthy functional endothelium is of outmost importance against vascular disease. In this study, human induced pluripotent stem (iPS) cells were reprogrammed from 1 ml blood of healthy donors and subsequently differentiated into endothelial cells (iPS‐ECs) with typical EC characteristics. This research combined iPS cell technologies and next‐generation sequencing to acquire an insight into the transcriptional regulation of iPS‐ECs. We identified endothelial cell‐specific molecule 1 (ESM1) as one of the highest expressed genes during EC differentiation, playing a key role in EC enrichment and function by regulating connexin 40 (CX40) and eNOS. Importantly, ESM1 enhanced the iPS‐ECs potential to improve angiogenesis and neovascularisation in in vivo models of angiogenesis and hind limb ischemia. These findings demonstrated for the first time that enriched functional ECs are derived through cell reprogramming and ESM1 signaling, opening the horizon for drug screening and cell‐based therapies for vascular diseases. Therefore, this study showcases a new approach for enriching and enhancing the function of induced pluripotent stem (iPS) cell‐derived ECs from a very small amount of blood through ESM1 signaling, which greatly enhances their functionality and increases their therapeutic potential. Stem Cells *2019;37:226–239*

**Significance Statement**

In regenerative medicine and cell therapy, endothelial cell (EC) reprogramming is a major tool for understanding key factors in treating vascular disease. Thus, facilitating the function and maintenance of generated ECs is very important. The current study has revealed, for the first time, the role of ESM1 signaling in improving the function and neovascularization potential in ECs generated from induced pluripotent stem cells in vitro and in vivo.

## Introduction

The complications arising from macrovascular and microvascular disease can be a major source of mortality in diseases such as diabetes and cardiovascular disease 1, 2. As a result, removing the causative insult is one of the primary strategies to repair endothelial cells (ECs) in vivo. However, when this approach is not adequate another important strategy is vascular regenerative medicine, which could repair and regenerate damaged cells including the generation of functional ECs for transplantation 3.

Induced pluripotent stem (iPS) cell‐derived endothelial cells (iPS‐ECs) have shown notable therapeutic potential in preclinical studies, which includes the ability to incorporate into and re‐endothelialize damaged vasculature 3, 4 as well as to inhibit neointimal and inflammatory responses to vascular injury 5. In addition, they have shown great functional promise in providing opportunities for disease modeling 6, 7. Consequently, iPS cells in regenerative medicine show great potential today as they can be used to generate patient‐specific cells and personalized therapies 8.

Even though there are many approaches in reprogramming methodology 9, 10, many of the iPS cell generation mechanisms and their subsequent differentiation toward various cell lineages still remain relatively unclear. Moreover, prolonged culture of iPS‐ECs populations can limit their clinical application and since they are not derived from intact blood vessels, they show an immature phenotype 11, 12. iPS‐ECs generation efficiency has increased over the last years, resulting in nearly pure populations of endothelial‐like cells 13. However, these methods still present several limitations such as population heterogeneity transient endothelial phenotype and differentiation variability between cell lines. Recent studies have, thus, attempted to improve the aforementioned issues by improving the protocols for EC generation 14 and refining the acquisition of purer iPS‐ECs populations through fluorescence‐activated cell sorting (FACS) 15 or EC progenitors through magnetic activation cell sorting (MACS) 16, 17. However, more effort still needs to be made toward the understanding of the mechanisms that drive iPS‐ECs differentiation in a well‐defined and highly reproducible environment.

In the pursuit to understand the underlying mechanisms of the EC differentiation process, this research combined iPS cell technologies and next‐generation sequencing to acquire a comprehensive insight into the transcriptional regulation of iPS‐ECs differentiation. More specifically, the ultimate goal of this work was to identify endothelial lineage‐enriched genes that have the potential to eventually increase iPS‐ECs enrichment. To achieve that, human iPS cells were reprogrammed from 1 ml of blood using nonintegrating reprogramming vectors from healthy donors in 7–10 days based on a fast and highly efficient approach. Up to six different donors were used to establish iPS cell lines for this study, which were then differentiated into iPS‐ECs displaying typical EC characteristics. Subsequently, high‐throughput RNA‐Sequencing (RNA‐Seq) of iPS cells, iPS‐ECs, and a subset of primary endothelial cells (human umbilical vein endothelial cells [HUVECs]) was implemented to undertake comparative transcriptome analyses. Out of the many genes, endothelial cell‐specific molecule 1 (ESM1) emerged as a primary candidate due to its known implication in a variety of cell functions such as angiogenesis 18 and EC responses due to stress factors and disease 19. As a result, we found that the Endothelial Cell‐Specific Molecule 1 (ESM1) holds a key role in enrichment and improved function of iPS‐ECs.

## Materials and Methods

Cell culture media, serum, and cell culture supplements were purchased from ATCC (Manassas, VA, USA), Merck Millipore (Billerica, MA, USA), LONZA, Basel, Switzerland, Sigma–Aldrich (St. Louis, MO, USA), Becton Dickinson Biosciences, New Jersey, USA, Reprocell, Yokohama, Japan, and Thermo Fisher Scientific (Waltham, MA, USA). Human aortic endothelial cells (HAoECs) were bought from Brennan and Co. (Dublin, Ireland) via PromoCell, Ireland (C‐12271). Magnetic beads were purchased from Miltenyi Biotec (Bergisch Gladbach, Germany). Antibodies against ESM1 (ab103590), mCherry (ab125096), TRA‐1‐60 (ab16288), Lin28 (ab46020), eNOS (ab76198), KLF4 (ab72543) were purchased from Abcam (Cambridge, U.K.). CD144 (VE‐Cadherin; ab33168 and STJ96234) and Oct4 (ab19857 and STJ72238) were purchased from Abcam or St. John's Laboratory (London, U.K.). Connexin‐40 (STJ96742) was purchased from St. John's Laboratory. Antibodies against vWF (SC‐8068) were purchased from Santa Cruz Biotechnology Inc. (Santa Cruz, CA, USA). VEGFR2 (MAB3571) and β‐actin (MAB8929) were purchased from R&D Systems Inc. (Minneapolis, MN, USA). ZO‐1 (40‐2200) was purchased from Invitrogen (Carlsbad, CA, USA). CD31/PECAM‐1 (ab28364 and BBA7) was purchased from Abcam and R&D, while GAPDH (sc‐25778 and ab8245) was purchased from Santa Cruz and Abcam. The secondary antibodies for immunostaining anti‐mouse Alexa 568, and anti‐rabbit Alexa 488, anti‐rabbit Alexa 568, anti‐goat Alexa 568, anti‐goat alexa 488 were purchased from Thermo Fisher Scientific. The secondary antibodies for Western blotting were purchased from Abcam and Cell Signaling.

### Blood Mononuclear Cells Isolation and Expansion

In this study, iPS cell lines have been generated from at least six donors. Written informed consent was obtained from each donor before blood collection. Nonmobilized peripheral blood (1–20 ml) was collected by venepuncture in EDTA‐coated 4 ml tubes. The blood was gradient‐separated by layering it on Histopaque solution (1:1 ratio) and spinning for 30 minutes at 550gx at room temperature (break OFF). The mononuclear cells (MNCs) formed a buffy coat between the plasma layer and the Histopaque buffer layer, and were collected using a soft plastic pipette. After three washes with phosphate buffered solution (PBS), the cells were resuspended in 1 ml of MNC medium and plated at a density of 4 million cells/ml. After 7 days of expansion, changing the medium every 3 days, the cells were either cryopreserved or subjected to iPS cells reprogramming and EC differentiation (see also Supporting Information Appendix).

### RNA Sequencing

Cells were briefly washed with PBS and harvested using QIAzol lysis buffer. Total mRNA and miRNA was extracted and purified using miRNAeasy (QIAGEN). The RNA concentration was determined using NanoDrop spectrophotometer and the integrity of the sample was assessed using Agilent RNA 6000 Nano Kit and Agilent 2100 Bioanalyzer (Agilent Technologies, Palo Alto, CA). RNA sequencing libraries were prepared using a stranded KAPA RNA‐Seq Kit with RiboErase (KapaBiosystems, Wilmington, MA, USA) according to the manufacturer's instructions with 1 μg total RNA in 10 μl RNase‐free water as an input. Libraries were sequenced on a NextSeq (Illumina) and reads mapped to the human reference genome (hg38), allowing up to 2 mismatches and up to 10 hits per read using the CLC Genomics Workbench 10.0.1 (https://www.qiagenbioinformatics.com). Reference sequences were annotated with genes and transcripts. Reference content was mapped to gene regions only. Expression values per gene or transcript were defined by total counts. A false discovery rate (FDR)‐adjusted *p*‐value of ≤.05 was chosen to indicate statistical significance. Comparative gene expression data was filtered according to the defined fold change and FDR‐adjusted *p*‐value thresholds. Statistical analysis results were displayed as volcano plots, where −log_10_(*p*‐values) are plotted against log_2_(fold change) for each feature. Hierarchical clustering of features (Manhattan distance; single linkage) was used to identify genes with similar expression patterns over the samples. K‐mean clustering was also performed (number of partitions = 5; distance metric = Manhattan distance). For all RNA sequencing steps implemented in CLC, default settings were used, unless stated otherwise. Detailed methods and materials can be found in Supporting Information Appendix and Experimental Procedures.

## Results

### Efficient Generation of Integration‐Free iPS Cells from Peripheral Blood MNCs in 9 Days

**Figure 1:**
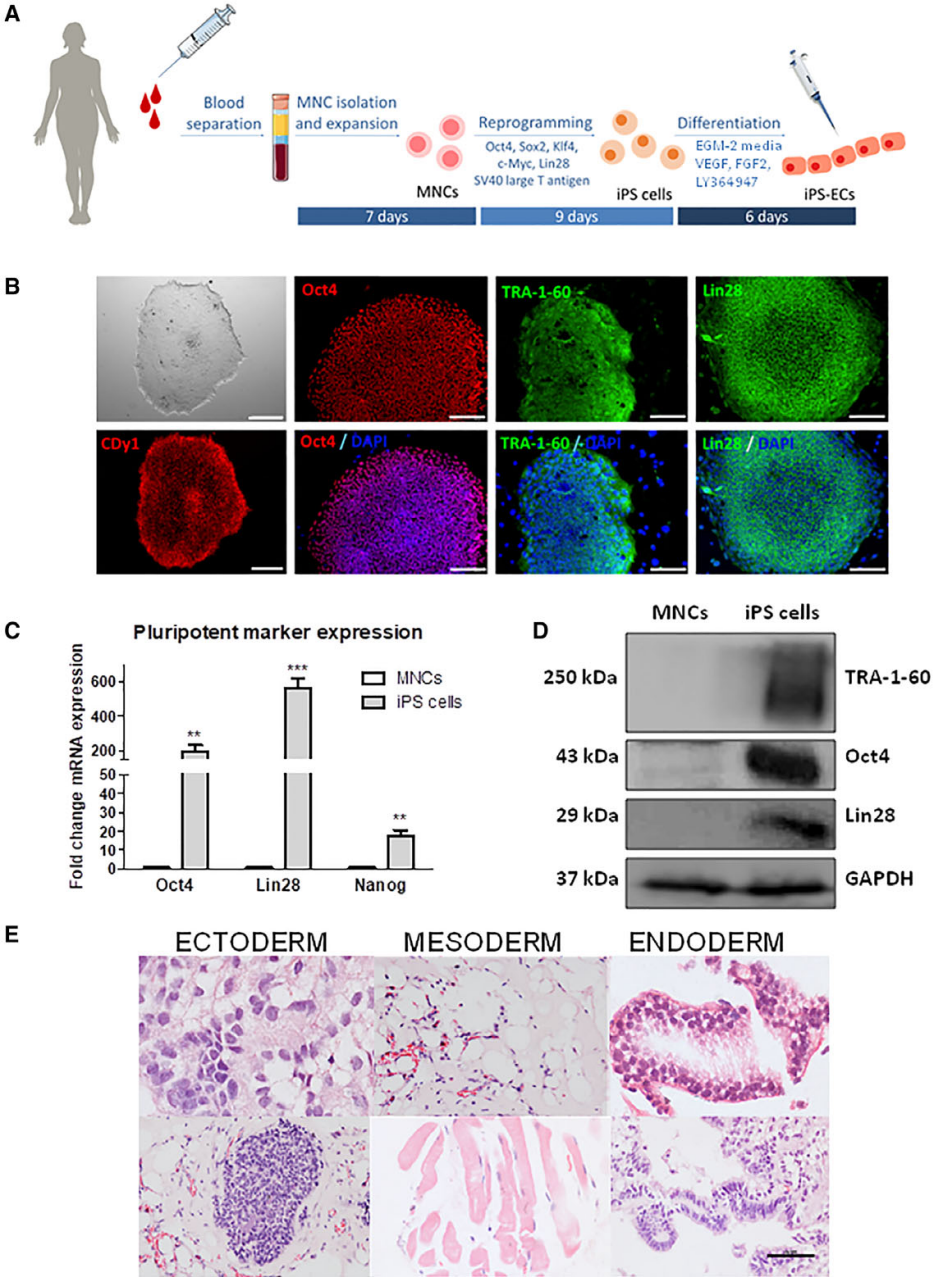
Efficient generation of integration‐free induced pluripotent stem (iPS) cells from peripheral blood mononuclear cells in 9 days. **(A):** Schematic flow diagram depicting the flow from 1 ml blood sample to iPS cells and iPS‐endothelial cells (ECs). **(B):** Phase contrast image showing the typical appearance of iPS cells colonies. Immunofluorescence assay for pluripotency markers Oct4, TRA‐1‐60, Lin28, and CDy1. Nuclei are counterstained with DAPI. Scale bars are 100 μm except for the left images where they are 200 μm. **(C):** Real‐time PCR showing the mRNA expression levels of Oct4, Lin28, and Nanog in mononuclear cells (MNCs) and iPS cells. Data were normalized to GAPDH (data are means ± SEM [*n* = 3]; **, *p* < .01; ***, *p* < .001). **(D):** Western blot showing TRA‐1‐60, Oct4, and Lin28 protein expression in MNCs and iPS cells. GAPDH was used as a loading control to correct for protein loading. **(E):** Hematoxylin and eosin (H&E) staining of iPS cells‐associated teratoma formation in vivo following subcutaneous injection into severe combined immunodeficiency mice. The data presented are representative or means (±SEM) of three independent experiments.

MNCs were isolated from healthy donor peripheral blood and expanded for 7 days before being successfully reprogrammed into iPS cells (Fig. 1A). MNCs were initially isolated from a starting amount of 20 ml of blood, followed by a scaling down procedure to 1 ml of blood. Because the efficiency of MNC reprogramming is lower than reprogramming from other cell sources, instead of only using the standard Yamanaka factors 20, we used the nonintegrating episomal plasmid vectors pEB‐C5 (overexpressing Oct4, Sox2, Klf4, c‐Myc, and Lin28), and pEB‐Tg vector (overexpressing SV40 large T antigen) 21, 22 to generate iPS cells in a fast and robust manner. Around day 9, typical iPS cell colonies with well‐defined round limits were observed (Fig. 1B). Established iPS cell colonies from 1 ml of blood were determined to be pluripotent through the assessment of extensively characterized pluripotency‐associated markers. iPS cell colonies stained positive for Oct4, TRA‐1‐60, Lin28, and CDy1 (Fig. 1B). Additional characterization using real‐time polymerase chain reaction (PCR) (Fig. 1C) and immunoblotting (Fig. 1D) confirmed that iPS cells exhibit markedly enhanced expression of pluripotency markers at mRNA and protein levels. MNCs did not express these markers (Fig. 1C, 1D). Lastly, teratoma formation is widely accepted as the gold standard for defining bona‐fide iPS cells. Following subcutaneous injection into severe combined immunodeficiency (SCID) mice, iPS cells formed tumoural structures with components of all three germ cell layers—ectoderm, mesoderm, and endoderm (Fig. 1E). Taken together, this fast and robust 9‐day protocol successfully reprogrammed donor MNCs from as little as 1 ml of blood to fully pluripotent iPS cells using nonintegrating methodology, whose pluripotent characteristics were confirmed on a number of levels.

### Differentiation of Human iPS Cells to iPS‐ECs

**Figure 2:**
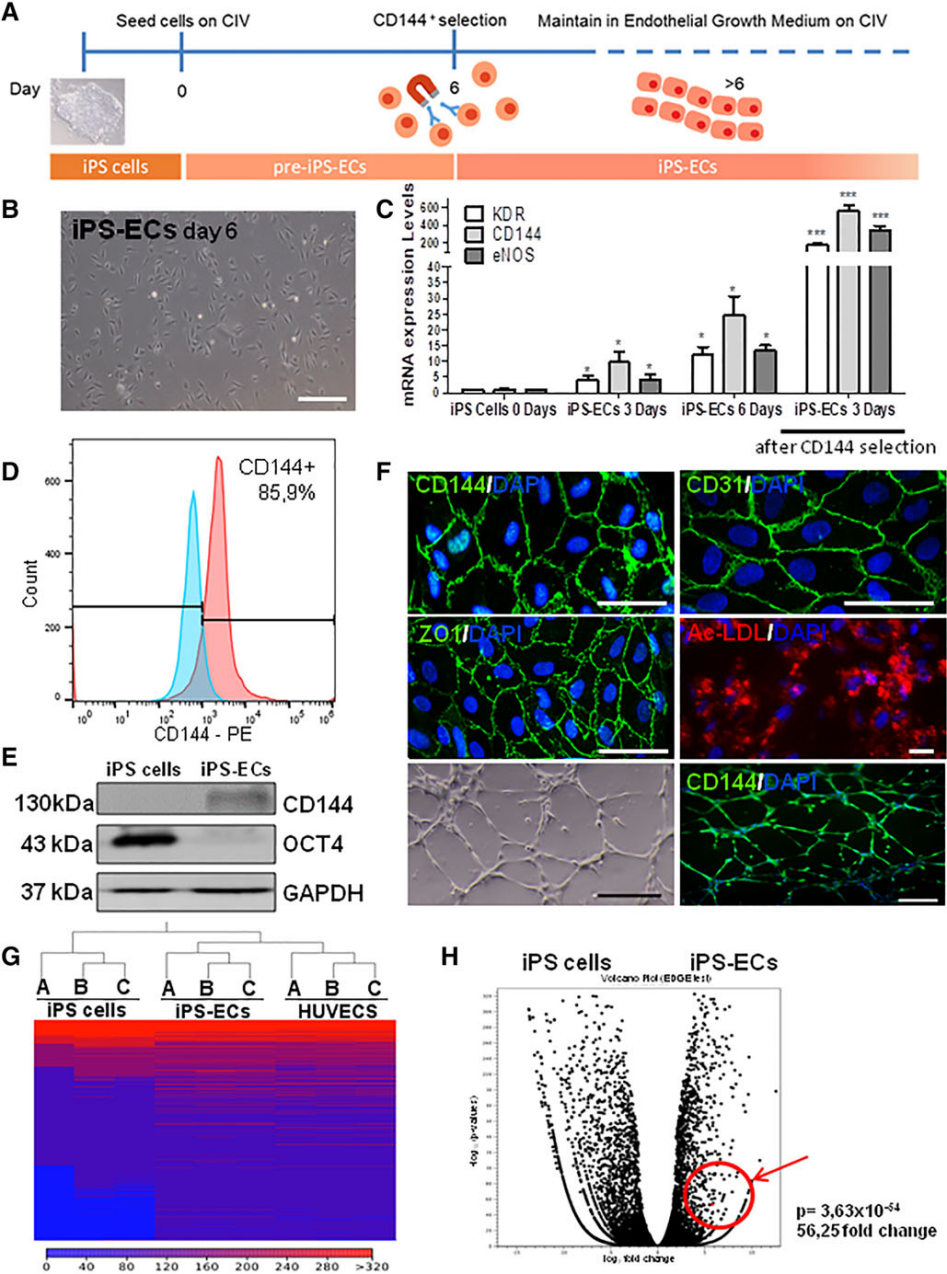
Differentiation of human induced pluripotent stem (iPS) cells to iPS‐endothelial cells (ECs). **(A):** Schematic diagram depicting the protocol for differentiating iPS cells to iPS‐ECs. **(B):** Phase contrast imaging showing the characteristic morphology of iPS‐ECs. Scale bar = 200 μm. **(C):** Real‐time PCR showing how mRNA expression levels for the endothelial markers KDR, CD144, and eNOS change in iPS‐ECs during endothelial differentiation from day 0 to 6 and 3 days after CD144 selection (data are means ± SEM [*n* = 3]; *, *p* < .05; ***, *p* < .001). **(D):** Flow cytometry analysis showing the percentage of cells that express CD144 before selection compared with cells stained with isotype control. **(E):** Western blot showing the protein expression of CD144 and Oct4 in iPS cells and iPS‐ECs after differentiation and CD144^+^ selection. **(F):** Immunofluorescent staining for CD144 (top left), CD31 (top right), and ZO‐1 (middle left). Junctional markers are stained green. Nuclei are counterstained with DAPI (blue). Images depicting the functional qualities of iPS‐ECs—acetylated (ac)‐LDL uptake (red; middle right), in vitro tube formation assay on Matrigel (bottom left) and immunofluorescent staining for CD144 (green) in in vitro capillary‐like structures (bottom right). Nuclei are counterstained with DAPI (blue). Scale bars are all 50 μm except for the bottom images, where it is 200 μm. **(G):** Comparison of overall gene expression profiles for iPS cells, iPS‐ECs, and human umbilical vein endothelial cells (HUVECs): heat map (Manhattan distance, single linkage) showing hierarchical clustering results for iPS cells, iPS‐ECs, and HUVECs replicates. Normalized expression values were used for hierarchical clustering. In this heat map, red represents genes that are more highly expressed within a given cell line, while blue represents genes that are expressed at lower levels within a defined cell line. (A), (B), and (C) refer to group replicates. **(H):** Volcano plot of differentially expressed genes in iPS cells versus iPS‐ECs depicting statistical significance as log10 (*p*‐values) on the *y*‐axis plotted against fold change as log2 (fold change) on the *x*‐axis. Genes with greater expression in iPS‐ECs are plotted on the right side of the plot. The data presented are representative or means (±SEM) of three independent experiments.

To differentiate iPS cells to iPS‐ECs, cells were seeded on collagen IV‐coated plates in EGM‐2 media with bone morphogenetic protein 4 (BMP4), Activin A, 6‐[[2‐[[4‐(2,4‐dichlorophenyl)‐5‐(5‐methyl‐1H‐imidazol‐2‐yl)‐2‐pyrimidinyl]amino]ethyl]amino]‐3‐pyridinecarbonitrile (CHIR99021), and fibroblast growth factor 2 (FGF2). As it was expected, undifferentiated iPS cells were negative for EC markers such as CD31 prior to EC differentiation as flow cytometry data showed in Supporting Information Figure S1A. After 48 hours, the medium was supplemented with vascular endothelial growth factor (VEGF), FGF2, and 4‐[3‐(2‐pyridinyl)‐1H‐pyrazol‐4‐yl]‐quinoline (LY364947) and was refreshed every other day. On day 6, positive selection of cells mediated by MACS was performed, according to the expression of the endothelial‐specific cell surface marker CD144, the most specific EC marker, and the cells were expanded and used for the experiments. From this point onward, iPS‐ECs positive for CD144 were cultured in conditions that would maintain the endothelial phenotype in the presence of VEGF and LY364947. The CD144 negative population was briefly characterized showing positive expression of the stromal marker CD90 (Supporting Information Fig. S1C). In essence, this 6‐day EC differentiation protocol (Fig. 2A) is both efficient and effective in generating iPS‐EC lines. Following EC differentiation, the morphological appearance of the cells progressed away from the three‐dimensional organization of iPS cells to a flatter, more cobblestone‐like morphology (Fig. 2B). In comparison to iPS cells, iPS‐ECs displayed upregulation of the EC markers KDR, CD144, and eNOS at the mRNA level (Fig. 2C), confirming their progression toward the vascular lineage. This upregulation was progressive between days 0 and 6 of differentiation (Fig. 2C), while a decline was observed on day 9 (Supporting Information Fig. S1B). Since there was a peak of EC marker expression on day 6 of differentiation and more than 80% of the cells were CD144^+^ (Fig. 2D), we performed MACS‐mediated selection of CD144^+^ cells on day 6 and named the positively selected cells as iPS‐ECs from this point on. Additional real‐time data also confirmed high levels of EC markers 3 days after CD144 selection (Fig. 2C). After this point, the cells were used for experiments and further immunoblotting experiments confirmed that, at the protein level, iPS‐ECs had upregulated CD144 with concomitant downregulation of Oct4 (Fig. 2E). A key feature of mature ECs is their ability to form junctions. Such junctions permit adhesion and communication between ECs and as such, are involved in regulating processes including paracellular permeability, cell growth and angiogenesis 23. Indeed, immunofluorescent staining of iPS‐ECs confirmed their ability to form adherens and tight junctions, as evidenced by specific staining for CD144, CD31, and ZO‐1 (Fig. 2F). iPS‐ECs were positively stained using a low‐density lipoprotein uptake assay, in which cells took up Acetylated (Ac)‐LDL (Fig. 2F). When seeded onto Matrigel, iPS‐ECs formed capillary‐like structures in vitro, which were positively stained with CD144 (Fig. 2F). Overall, this EC differentiation protocol provides a robust method to produce populations of iPS‐ECs.

### A Genome‐Wide Shift Away from Pluripotency

To gain a detailed insight into the molecular changes that occur following the EC differentiation of iPS cells, RNA‐seq was performed. Gene expression profiles were compared for iPS cells, iPS‐ECs, and HUVECs in at least three independent experiments. HUVECs were chosen as representative primary ECs. Generated RNA‐Seq outputs were analyzed using the CLC Genomics Workbench 10.0.1 (https://www.qiagenbioinformatics.com) to identify differential expression patterns. Count distributions were visualized using box plots. In line with data quality assessment, expression values were normalized. To explore similarities and differences between cell lines, hierarchical clustering of samples was undertaken, which indicated that replicates clustered together, as expected (Fig. 2G). Of note, iPS‐ECs clustered more closely with HUVECs than with iPS cells, confirming that the transcriptional network pertaining to these stem cell‐derived ECs has undergone a shift in expression profile away from pluripotency and toward a specialized EC population. A scatterplot depicts the overall gene expression changes between iPS cells and iPS‐ECs (Supporting Information Fig. S2A). To confirm that iPS‐ECs were a committed cellular population, fold change values for key pluripotency markers were assessed. Their genetic shift away from pluripotency was evidenced by the significant downregulation of Nanog (−2,731; *p* = .00), Oct4 (−23,009; *p* = .00), Lin28A (−10,073; *p* = .00), and SOX2 (−71; *p* = 1.06 × 10^−25^) in iPS‐ECs versus iPS cells (Supporting Information Fig. S2B). This confirms that the differentiation protocol implemented here successfully generated EC‐like cells from iPS cells by overwriting the pluripotent profile of the starter cell population. Importantly, iPS‐ECs’ transcriptomes reflect expression patterns that are closer to that of primary mature ECs.

### EC Differentiation Enriches for Key Endothelial Characteristics and ESM1 Signaling

To obtain a more detailed insight into the endothelial qualities of iPS‐ECs, differential expression patterns for EC lineage‐specific markers were assessed. iPS‐ECs displayed a unique gene expression profile that separated them from iPS cells. When analyzing differential expression, data was filtered to include only genes that were differentially expressed at a defined significance level. To remove genes with low fold change values, a fold change threshold of ≥2 was considered to indicate differential expression. An FDR‐adjusted *p*‐value of ≤.05 indicated statistical significance. Under these parameters, 3,495 genes were significantly upregulated in iPS‐ECs compared with iPS cells. When compared with iPS cells, sequencing confirmed that iPS‐ECs displayed upregulation of endothelial markers (Supporting Information Fig. S3A, S3B). iPS‐ECs also demonstrated significant upregulation of EC‐specific signaling (Supporting Information Fig. S3A, S3B). Interestingly, a number of genes that were upregulated in iPS‐ECs, including PTX3 and GDF5 are known to be related with angiogenesis 24, 25. Importantly, the generated iPS‐ECs population appears to be heterogeneous containing both arterial markers, such as NRP1 and EPHB2, as well as, the venous marker, NR2F2 (Supporting Information Fig. S3A, S3B). However, higher expression of arterial markers indicates a greater tendency toward this subtype. Gene functional classification was also undertaken to identify groups of related genes that were enriched in iPS‐ECs versus iPS cells. In the resting state ECs are efficient antigen‐presenting cells. Correspondingly, iPS‐ECs demonstrated functional enrichment for various membrane protein‐encoding genes (Supporting Information Fig. S4). Annotation with GO “Biological Process” terms (Supporting Information Fig. S5A) confirmed enrichment for characteristic vascular processes including vascular development (*p* = 1.45 × 10^−6^), blood vessel morphogenesis (*p* = 9.08 × 10^−6^) and regulation of cell adhesion (*p* = 9.71 × 10^−6^; Supporting Information Fig. S5A). These processes identified enriched genes such as ENG, a novel EC specification gene 26 and FOXF1, a critical transcription factor that regulates embryonic vasculature development 27. Enrichment patterns, therefore, correlate with the ability of iPS‐ECs to form vascular structures. Furthermore, annotation of data with GO “Cellular Component” terms revealed enrichment for genes located in the extracellular region of the cell, including genes with roles in EC lineage specification and function (Supporting Information Fig. S5B). Examples included FGF5, TGF‐β1, ANGPT2, and ESM1. Based on these analyses, iPS‐ECs possess transcriptomic profiles that will allow them to engage in a vast array of endothelial‐related functions. Interestingly, the RNA‐Seq analysis has revealed a high expression level of the gene ESM1 in iPS‐ECs compared with iPS cells, as the Volcano plot shows in Figure 2H. As such, ESM1 held the potential to be an important candidate implicated in angiogenesis. Therefore, this study has further focused on the underlying mechanisms and function of ESM1 in iPS‐ECs, with the ultimate goal to enrich the function of the derived iPS‐ECs based on a fully defined environment.

### ESM1 Regulates EC Marker Expression in iPS‐Derived ECs

**Figure 3:**
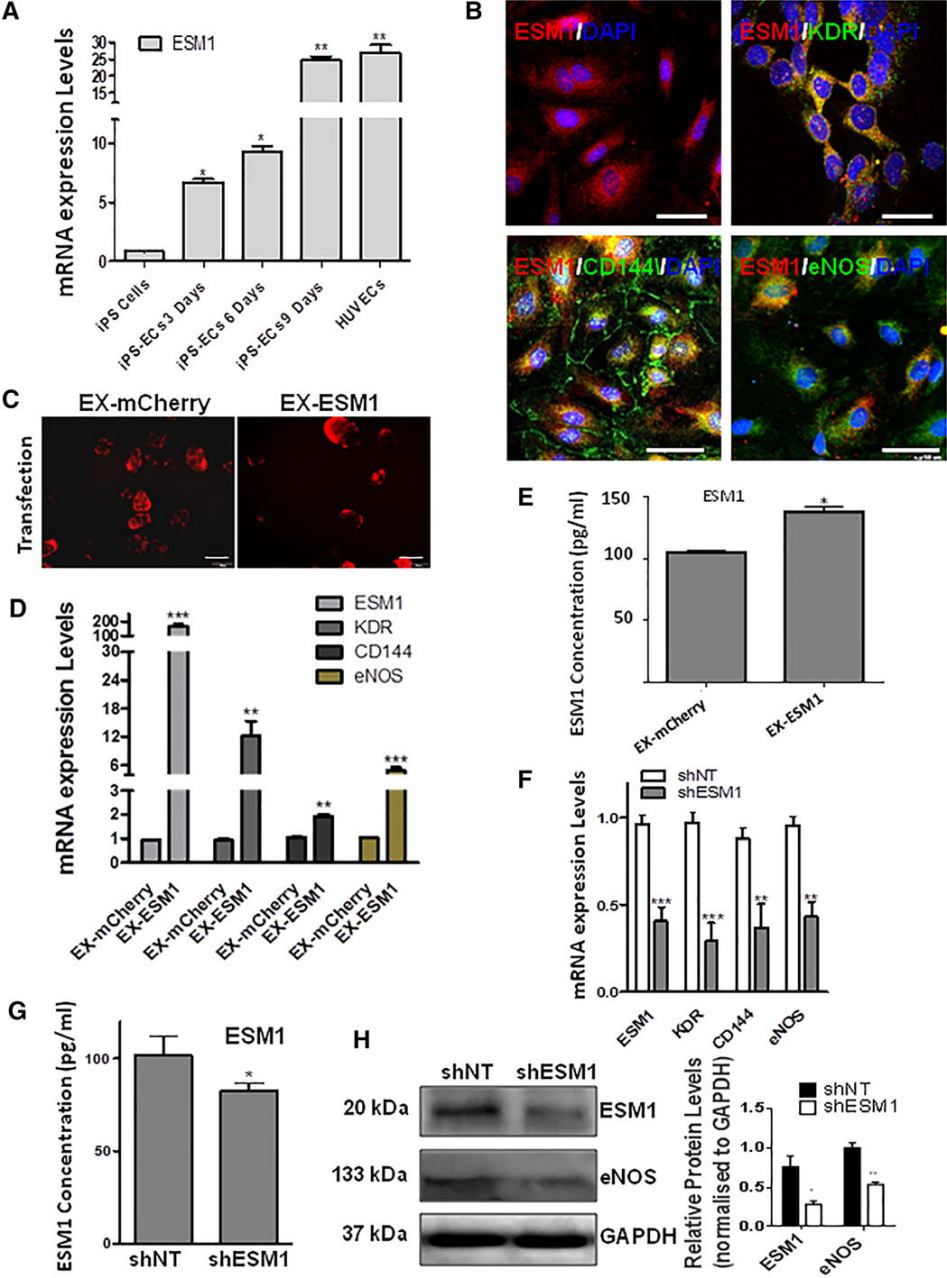
ESM1 regulates endothelial cell (EC) marker expression in ECs from induced pluripotent stem (iPS) cells and ESM1 signaling. **(A):** Real‐time PCR data showing comparison of ESM1 mRNA expression levels between iPS cells, iPS‐ECs (at 3, 6, and 9 days of EC differentiation), and human umbilical vein endothelial cells (HUVECs; data are means ± SEM [*n* = 3]; *, *p* < .05; **, *p* < .01). **(B):** Immunofluorescent images showing costaining of ESM1 (red), EC markers KDR, eNOS, CD144 (green), and DAPI (blue). Scale bars are 50 μm. **(C):** Representative immunofluorescent images of iPS‐ECs overexpressing EX‐mCherry (red) and EX‐ESM1 (red) 48 hours after transfection with the corresponding plasmids. Scale bars are 100 μm. **(D):** Forty‐eight hours after ESM1 overexpression, a significant increase in mRNA expression of EC markers KDR, CD144, and eNOS was observed (data are means ± SEM [*n* = 3]; **, *p* < .01; ***, *p* < .001). **(E):** ESM1 protein concentration levels 48 hours after ESM1 overexpression were significantly increased in the cell culture media compared with control, as detected by Luminex assay (data are means ± SEM [*n* = 3]; *, *p* < .05). **(F):** ESM1 knockdown 72 hours after lentiviral transduction with shESM1, compared with nontargeting control (shNT), resulted in significantly decreased mRNA levels of ESM1 and EC markers KDR, CD144, and eNOS (data are means ± SEM [*n* = 3]; **, *p* < .01; ***, *p* < .001). **(G):** ESM1 protein concentration levels 72 hours after ESM1 knockdown were significantly decreased in the cell culture media compared with control, as detected by Luminex (data are means ± SEM [*n* = 3]; *, *p* < .05). **(H):** Western blot (left panel) and corresponding densitometry (right panel) showing decreased protein levels in ESM1 and eNOS in iPS‐ECs with ESM1 knockdown. The data presented are representative or means (±SEM) of three independent experiments (data are means ± SEM [*n* = 3]; *, *p* < .05; **, *p* < .01).

To validate the RNA‐Seq data and explore whether ESM1 is implicated in EC enrichment, real‐time PCR was performed to monitor expression levels of ESM1 mRNA in iPS cells, differentiating iPS‐ECs at 3, 6, and 9 days of EC differentiation, and HUVECs. ESM1 mRNA levels significantly increased over time in iPS‐ECs compared with the control iPS cells, reaching a peak at 9 days of differentiation, the levels of which were comparable to HUVECs (Fig. 3A). Seeing these results, we wanted to assess the role of ESM1 on iPS‐ECs after differentiation and selection of CD144^+^ cells. When such iPS‐ECs were visualized using immunofluorescent microscopy, ESM1 costained with EC markers KDR, CD144, and eNOS, indicating their concurrent expression (Fig. 3B). ESM1 was cloned in an overexpression vector‐tagged with mCherry (EX‐ESM1) which was used to overexpress ESM1 in iPS‐ECs (Fig. 3C). Strikingly, significant increase in mRNA expression of EC markers KDR, CD144, and eNOS was observed 48 hours after transfection in mRNA level (Fig. 3D). In addition, ESM1 secretion levels, as detected by Luminex assay, were significantly increased in the cell culture media 48 hours after ESM1 overexpression compared with controls (Fig. 3E). Seventy‐two hours after ESM1 knockdown by lentiviral transduction, the mRNA levels of ESM1 and EC markers KDR, CD144, and eNOS were significantly decreased (Fig. 3F). This was also reflected in the cell culture media, in which significantly decreased ESM1 secretion levels were detected by Luminex (Fig. 3G). In addition, immunoblotting showed decreased protein levels in ESM1 and eNOS 72 hours after ESM1 knockdown (Fig. 3H), indicating its importance in regulating EC markers expression. Furthermore, support to the above notion is provided by additional data which shows that ESM1 overexpression in iPS‐ECs induced the expression of angiogenic signaling genes such as uPA, Endothelin‐1 and Angiopoietin‐2, with a concurrent downregulation of antiangiogenic factors (Supporting Information Fig. S6). Notably, ESM1 is also induced EC marker expression in early stages of EC differentiation (Supporting Information Fig. S7).

### ESM1 Regulates CX40 Expression in iPS‐ECs

**Figure 4:**
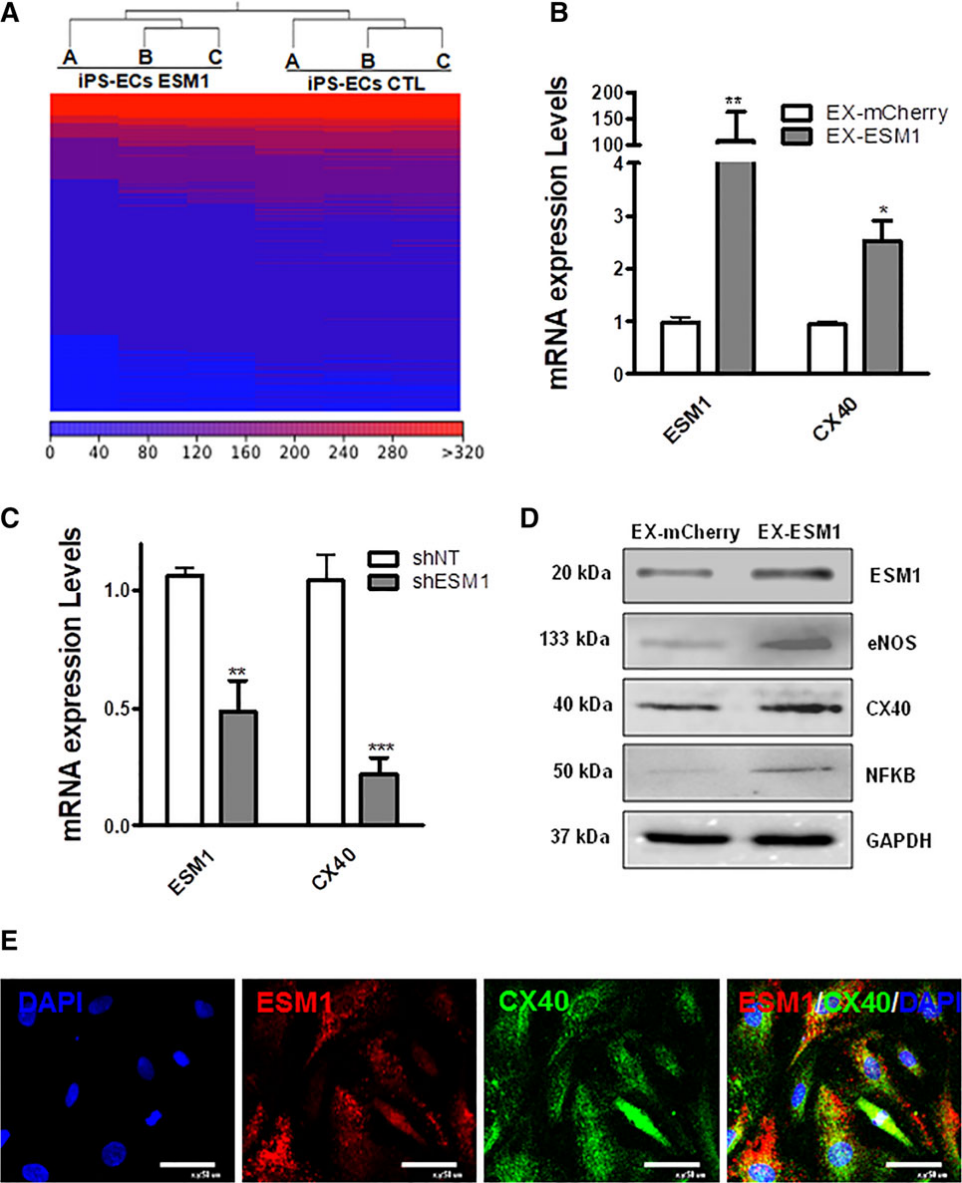
ESM1 regulates CX40 expression. **(A):** Heat map of RNA level differences between induced pluripotent stem‐endothelial cells (iPS‐ECs) overexpressing ESM1 (EX‐ESM1) compared with control iPS‐ECs (EX‐mCherry). **(B):** Overexpression of ESM1 leading to a significant increase in CX40 48 hours after transfection with EX‐ESM1 compared with control (EX‐mCherry; data are means ± SEM [*n* = 3]; *, *p* < .05; **, *p* < .01). **(C):** Knockdown of ESM1 leading to a significant decrease in CX40 72 hours after lentiviral transduction with shESM1 compared with non‐targeting control (shNT; data are means ± SEM [*n* = 3]; **, *p* < .01; ***, *p* < .001). **(D):** Western blots showing increased protein levels in ESM1, eNOS, CX40, and NFKB 48 hours after ESM1 overexpression. **(E):** Immunofluorescent images of cells costained with ESM1 (red), CX40 (green), and DAPI (blue). Scale bars are 50 μm. The data presented are representative or means (±SEM) of three independent experiments.

To shed further light into how ESM1 regulates EC markers, gene expression profiles were compared for iPS‐ECs overexpressing ESM1 (EX‐ESM1) and control iPS‐ECs overexpressing mCherry (EX‐mCherry). Generated RNA sequencing outputs were analyzed using the CLC Genomics Workbench 10.0.1 (https://www.qiagenbioinformatics.com) to identify differential expression patterns, as described above (Fig. 4A, and Supporting Information Fig. S8A, S8B). Interestingly, connexin 40 (CX40) was one of the highly upregulated genes in the next‐generation sequencing (NGS) in iPS‐ECs (EX‐ESM1) compared with control iPS‐ECs (EX‐mCherry; Fig. 4A, and Supporting Information Fig. S8A, S8B). In addition, iPS‐ECs when compared with aortic ECs revealed similar expression levels of the aortic marker Ephrin B2 and the concurrent expression of ESM1 and CX40 (Supporting Information Fig. S9). To confirm the RNA‐Seq data, ESM1 was overexpressed in iPS‐ECs, which resulted in significant increases in CX40 mRNA levels 48 hours after transfection with the EX‐ESM1 plasmid compared with control (EX‐mCherry; Fig. 4B). On the contrary, when ESM1 was knocked down, a significant decrease in CX40 mRNA expression was observed 72 hours after lentiviral transduction with shESM1 compared with nontargeting control (shNT; Fig. 4C). ESM1 overexpression in iPS‐ECs resulted in significant increases in protein levels of ESM1, eNOS, CX40, and nuclear factor‐kappa B (NFKB1) 48 hours after transfection with EX‐ESM1 plasmid compared with control (EX‐mCherry; Fig. 4D). When iPS‐ECs were visualized using immunofluorescent microscopy, ESM1 costained with CX40 indicating their concurrent expression (Fig. 4E).

### ESM1 Regulates EC Marker Expression in iPS‐ECs through CX40

**Figure 5:**
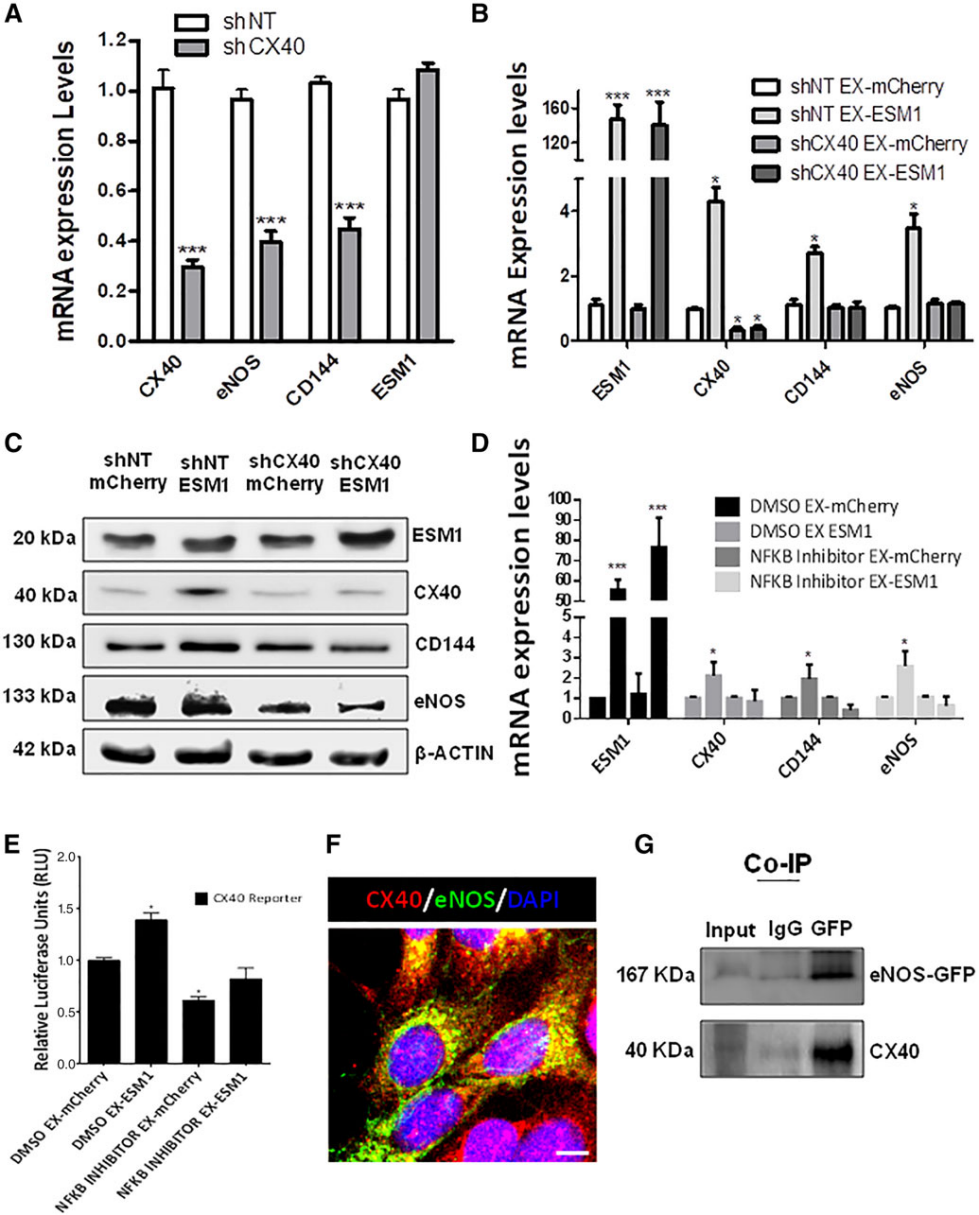
ESM1 regulates endothelial cells (EC) marker expression through CX40. Seventy‐two hours after lentiviral knockdown of CX40 (shCX40) in induced pluripotent stem (iPS)‐ECs, CX40, eNOS, and CD144 were significantly reduced at the mRNA level, but not ESM1 (data are means ± SEM [*n* = 3]; ***, *p* < .001). **(B, C):** CX40 was knocked down by shRNA on day 3 of EC differentiation, and ESM1 was overexpressed on day 4. (B) Real‐time PCR data and (C) Western blots reveal the induction of EC markers mediated by ESM1 is ablated by CX40 knockdown (data are means ± SEM [*n* = 3]; *, *p* < .05; ***, *p* < .001). **(D):** Cells were treated with NFKB inhibitor prior to ESM1 overexpression: CX40, CD144, and eNOS expression mediated by ESM1 was ablated after NFKB inhibition (data are means ± SEM [*n* = 3]; *, *p* < .05; ***, *p* < .001). **(E):** Luciferase assay for CX40 reporter in cells treated with NFKB inhibitor prior to ESM1 overexpression (data are means ± SEM [*n* = 3]; *, *p* < .05). **(F):** Immunofluorescent confocal image showing costaining of CX40 (red), eNOS (green), and DAPI (blue) in cells overexpressing eNOS‐GFP. Scale bars: 25 μm. **(G):** co‐IP showing CX40 and eNOS‐GFP interaction. The data presented are representative or means (±SEM) of three independent experiments.

ESM1 has been previously shown to increase the promoter activity and expression levels of NFKB 28, while putative transcription factor binding site research in the TRANSFAC database 29, 30 of the CX40 promoter revealed a binding site for NFKB. The above facts directed the notion that ESM1 induces the expression of CX40 in our cells possibly through NFKB induction. As a result, further experiments were performed to investigate how ESM1 regulates the expression of EC markers. Seventy‐two hours after lentiviral knockdown of CX40 (shCX40), CX40 and the EC markers eNOS and CD144 were significantly reduced at the mRNA level (Fig. 5A). In order to elucidate further the underlying mechanisms regulated by ESM1 in iPS‐ECs and explore the link with CX40, CX40 was knocked down using shRNA and ESM1 was overexpressed 24 hours later. Quantitative real‐time PCR data (Fig. 5B) and Western blots (Fig. 5C) revealed that the induction of EC markers mediated by ESM1 is ablated by CX40 knockdown, suggesting that in iPS‐ECs ESM1 regulates EC marker expression through CX40. When the cells were transfected with ESM1, followed by NFKB inhibitor treatment, EC marker expression was ablated in the treated cells compared with controls (Fig. 5D). In addition, Luciferase assay was performed to assess CX40 promoter activity after ESM1 overexpression and NFKB inhibition, revealing diminished activity levels compared with controls (Fig. 5E). Immunofluorescent confocal imaging confirmed the parallel expression of CX40 and eNOS (Fig. 5F and (Supporting Information Fig. S10A), whereas coimmunoprecipitation (Co‐IP) in cells stably expressing eNOS‐GFP, confirmed the CX40 and eNOS‐GFP interaction in (Fig. 5G). This data is in agreement with previous reports showing a role of CX40 in ECs in association with the EC marker eNOS 31, 32. Therefore, these experiments demonstrate that ESM1 regulates the expression of EC markers such as eNOS and CD144 through an association with CX40, an important EC gap junction channel component, possibly due to increased formation and stability of gap junctions, which are essential functional EC characteristics.

### ESM1 Induces Angiogenesis In Vivo

**Figure 6:**
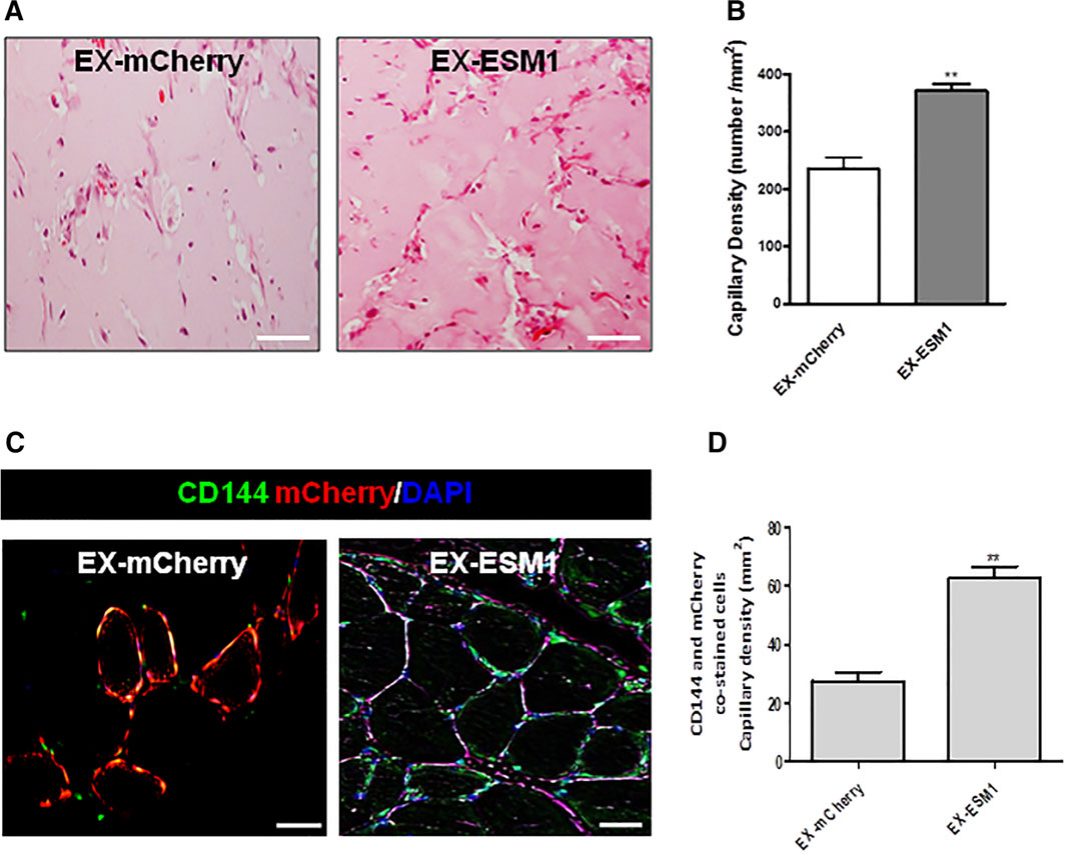
ESM1 improves angiogenesis and CD144 expression in vivo. Human induced pluripotent stem‐endothelial cells (iPS‐ECs) overexpressing either mCherry (EX‐mCherry) or ESM1 (EX‐ESM1) were injected subcutaneously in SCID mice 48 hours after transfection. **(A):** H&E staining of EX‐mCherry and EX‐ESM1 Matrigel plugs. EX‐ESM1 tissues significantly formed well‐defined vascular structures at 7 days in comparison to the control tissues where fewer vascular structures were observed. **(B):** Quantified capillary density (data are means ± SEM [*n* = 3]; **, *p* < .01). Quantification from 10 random microscopic fields at ×40, scale bars: 50 μm). Quantified capillary density expressed as capillary number per mm^2^. **(C):** Paraffin sections of CD144 immunofluorescent staining confirmed the presence of differentiated cells in the in vivo vascular tubes. **(D):** Quantified capillary density based on doubly stained CD144‐ and mCherry‐cells (data are means ± SEM [*n* = 3]; **, *p* < .01). Quantification from 10 random microscopic fields at ×40 (scale bars: 50 μm). Quantified capillary density expressed as capillary number per mm^2^. The data presented are representative or means (±SEM) of three independent experiments.

The next step was to validate the pivotal role of ESM1 in iPS‐ECs enrichment and function in vivo. Human iPS‐ECs were transfected with either EX‐mCherry plasmid or EX‐ESM1 plasmid. After 48 hours, 1 million iPS‐ECs overexpressing mCherry control (EX‐mCherry) or 1 million iPS‐ECs overexpressing ESM1 (EX‐ESM1) were injected subcutaneously in SCID mice. Hematoxylin and eosin (H&E) staining of the Matrigel plugs with EX‐mCherry and EX‐ESM1 revealed that EX‐ESM1 tissues formed defined vascular structures 7 days after the injection, in comparison to the control tissues where fewer vascular structures were observed (Fig. 6A, 6B). In addition, CD144 immunofluorescent staining of paraffin‐embedded tissue sections confirmed the presence of differentiated cells in the in vivo vascular tubes (Fig. 6C). The EX‐ESM1 tissues showed increased staining for CD144 and mCherry when compared with EX‐mCherry control tissues (Fig. 6D).

### ESM1 Significantly Improved Neovascularization and Blood Flow Recovery in the Hindlimb Ischemic Model

**Figure 7:**
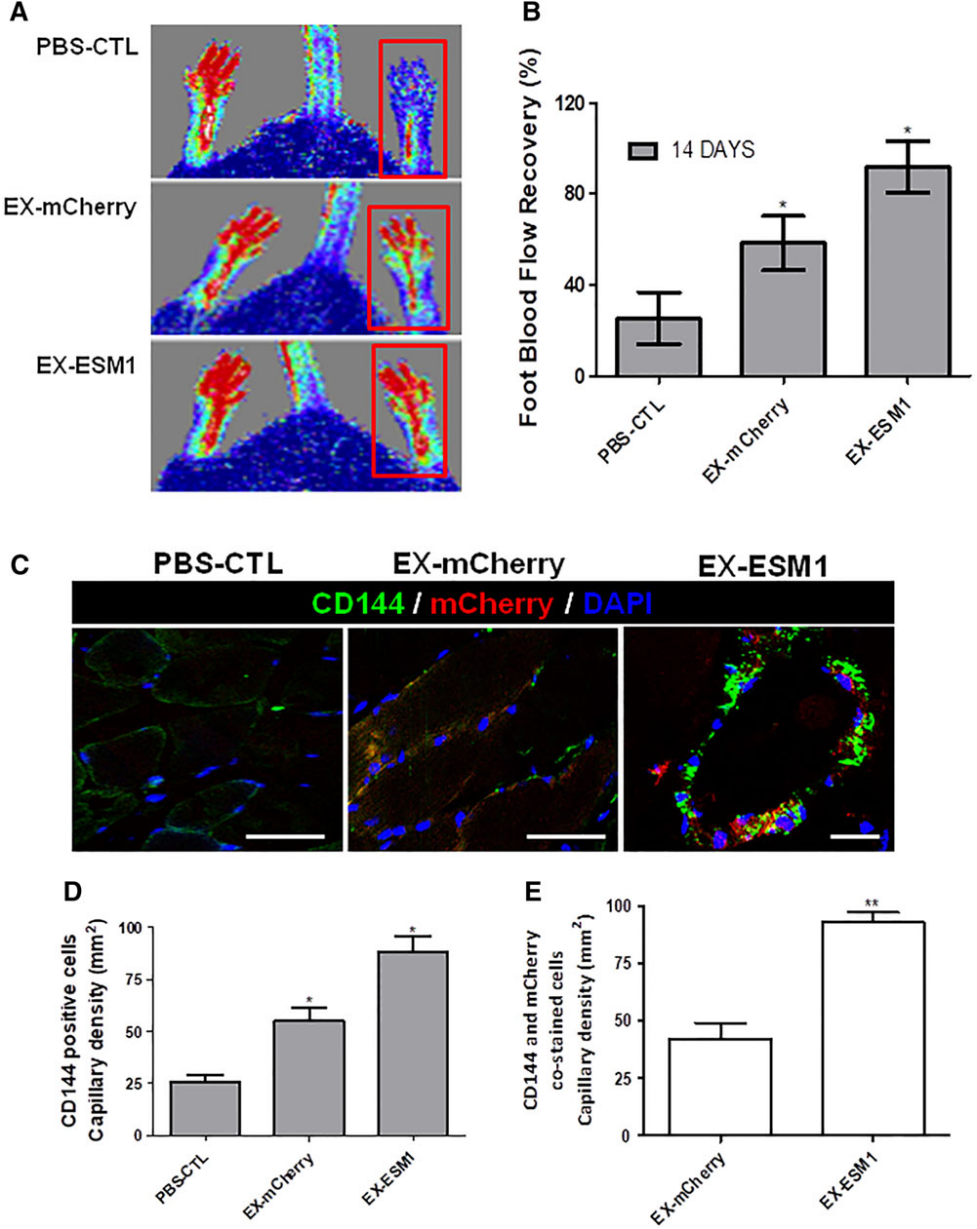
ESM1 significantly improved neovascularization and blood flow (BF) recovery in the hindlimb ischemic model. **(A):** Laser Doppler images of BF in the lower limbs of mice in prone position control after treatment with control induced pluripotent stem‐endothelial cells (iPS‐ECs; phosphate buffered solution (PBS)‐CTL or iPS‐ECs overexpressing EX‐mCherry) and iPS‐ECs overexpressing ESM1 (EX‐ESM1). **(B):** BF recovery in the ischemic foot (calculated as a percentage ratio between ischemic foot BF and the contralateral foot) for each of the conditions. Statistical analysis shows significantly higher BF recovery in the EX‐ESM1 mice at 14 days in comparison with controls; Bonferroni post‐test (to one way ANOVA) confirms significant difference after 14 days between the control iPS‐ECs and EX‐ESM1 (data are means ± SEM [*n* = 3]; *, *p* < .05). **(C):** Sections of adductor muscles of each condition were stained with CD144 antibody (left and middle scale bars are 50 μm, right scale bar is 100 μm) and capillary density was quantified and expressed as capillary number per mm^2^. **(D):** Data are means ± SEM (*n* = 3); *, *p* < .05. **(E):** Quantified capillary density of CD144 and mCherry doubly stained cells (data are means ± SEM; *n* = 3; **, *p* < .01). Quantification from 10 random microscopic fields at ×40. The data presented are representative or means (±SEM) of three independent experiments.

To supplement the findings from our in vitro and in vivo angiogenesis data and to confirm further in vivo relevance of EC enrichment function through ESM1 signaling, additional experiments were performed to investigate whether ESM1 could induce angiogenesis in ischemic tissues and improve blood flow (BF) recovery. iPS‐ECs (1 × 10^6^) overexpressing control mCherry (EX‐mCherry) or iPS‐ECs overexpressing ESM1 (EX‐ESM1) were injected intramuscularly into adductors of an ischemic model of SCID mice after induction of hindlimb ischemia in SCID mice, as we have previously reported 33. Laser Doppler images of BF in the lower limbs of mice in prone position 14 days postinjection of iPS‐ECS showed that the cells enhanced neovascularization and supported significantly higher BF recovery in the ischemic limbs compared with the PBS controls. Notably, EX‐ESM1‐injected mice showed even higher recovery than mice injected with EX‐mCherry cells (Fig. 7A, 7B). Furthermore, limbs receiving EX‐ESM1 cells displayed significantly higher capillary numbers in the musculature in comparison to their corresponding controls, as shown by positive staining of adductor muscle sections with CD144 (Fig. 7C) in immunopositive vessel profiles. Particularly, engrafted EX‐ESM1 cells displayed a typical and well‐defined vascular architecture (Fig. 7C, right panel). Finally, when adductor muscle sections from EX‐mCherry or EX‐ESM1 injected animals were stained and quantified for mCherry and CD144, EX‐ESM1 cells was found to exhibit an improved engraftment ability compared with controls (Fig. 7D, 7E), suggesting that EX‐ESM1 cells display improved characteristic endothelial functions in vivo. Altogether, our findings suggest that ESM1 overexpression induces vessel formation in vivo, further supporting its vital role in the enrichment of iPS‐ECs and improving their function, which can, in turn, enhance vascular network development.

## Discussion

Cellular therapy involving the transplantation of cells to replace or repair damaged vascular tissues and ECs is a highly esteemed regenerative approach toward improving vascular function in patients with ischemic disease. This current study has revealed, for the first time, the role of ESM1 in enriching human iPS‐ECs and enhancing their neovascularization potential both in vitro and in vivo*,* advancing our understanding surrounding their reprogramming and differentiation.

ESM1 protein, also known as endocan, is a dermatan sulfate proteoglycan 34 which is primarily expressed in ECs of vascular tissues including capillaries and arterioles but can also be found in the ECs of other tissues such as human lung and kidney 35. Its gene expression is regulated by cytokines, indicating its possible implication in endothelium‐based disorders 36. In addition, in recent studies, ESM1 has been identified as a specific biomarker of tip cells during angiogenesis 37 as well as a simultaneous target and modulator of VEGF signaling in ECs 38.

This study established an optimized protocol for the generation of iPS‐ECs from 1 ml of peripheral blood and used RNA‐Seq to acquire a fresh insight into the mechanisms that underlie the process of differentiation to ECs and the maintenance of the endothelial profile. It was hypothesized that this approach would allow the identification of novel specific genes and pathways that are key for the generation of high quality mature ECs. More specifically, gene expression profiles were compared for iPS cells, iPS‐ECs and HUVECs; iPS‐ECs clustered more closely with HUVECs compared with iPS cells, confirming a dramatic shift away from pluripotency toward a specialized EC population. In addition, this generated EC population expressed both arterial and venous markers, with a greater propensity for the former subtype, as evidenced by the presence of Ephrin B2. This would allow for translational application in arterial vessels, as well as functional enrichment for various protein‐encoding genes involved in vascular processes, development and blood vessel morphogenesis and EC lineage specification. Notably, using this model of iPS differentiation toward ECs, constitutive ESM1 expression was shown to be markedly upregulated during differentiation toward iPS‐ECs suggesting a possible crucial role in enhancing this process. Indeed, subsequent in vitro experiments using overexpression or knockdown of ESM1 confirmed its role in regulating key EC markers in iPS‐ECs.

A common limitation of iPS‐ECs is their restricted proliferative capacity, which is also often accompanied by augmented instability and senescence 39, creating a large barrier in the path toward their clinical application in treating vascular disease. It is, thus, imperative that we expand our understanding and, thus, identify more key factors that may facilitate faster and safer EC generation. In addition, differentiated ECs can shift their phenotype away from the endothelial lineage after prolonged cell culture and exhibit an immature phenotype 12, 40 with previous efforts focusing on preservation of EC stability and commitment 41, 42. Such observations of EC instability were also made in our study, in which ESM1 levels were reduced with increasing passages (after 15 passages; Supporting Information Fig. S10B). As a result, we demonstrated that ESM1 enhanced EC characteristics in iPS‐ECs. Notably, iPS‐ECs overexpressing ESM1 displayed greater functional properties in vitro and in vivo.

While investigating the mechanism by which ESM1 improved EC marker expression, it was noted that CX40 was significantly increased upon ESM1 overexpression. CX40 is a major connexin in most vascular ECs 43, a central component of gap junctions and important in communication 44 and harmonization of tissue responses 45, 46, 47.

In our study, further investigation on the relationship between ESM1 and CX40 revealed that CX40 acts downstream of ESM1. Particularly, we saw increased CX40 and amplified EC marker expression after ESM1 overexpression, which did not occur when we knocked down CX40. In agreement with our results, which showed increased EC marker expression and parallel expression of CX40 with eNOS, interaction of CX40 with eNOS has been shown to be important in the regulation of eNOS expression 31. Indeed, CX40 is central in endothelial intercellular communication and EC homeostasis as it interacts with eNOS and has been shown to be expressed in close proximity to eNOS at cellular interfaces in ECs 48. It has also been shown that ESM1 overexpression induces cell proliferation through induction of the NFKB pathway and that ESM1 interacts with NFKB and activates the NFKB promoter 28. Notably, transcriptional binding analysis of the CX40 promoter identified a binding site for NFKB, suggesting that ESM1 induces the expression of CX40 in iPS‐ECs, possibly through NFKB. In addition to the in vitro data, iPS‐ECs overexpressing ESM1 induced enhanced angiogenesis in vivo 7 days after subcutaneous injection in SCID mice and ESM1 overexpression in iPS‐ECs significantly improved neovascularization and BF recovery in the hindlimb ischemic model 14 days after intramuscular injection, demonstrating a crucial role of ESM1 in enhancing angiogenesis and neovascularization.

## Conclusion

In conclusion, our data noticeably demonstrate that iPS cell generation and further iPS‐ECs differentiation can be easily achieved using nonintegrating methodology from a small amount of blood, whilst also revealing a vital role for ESM1 in improving EC enrichment and function. More particularly, ESM1 enhances CX40 expression improving, in turn, the expression of EC markers such as eNOS in iPS‐ECs. The schematic diagram of the proposed mechanism is shown in the Graphical Abstract. This new approach for enhancing EC function of iPS‐ECs derived from a very small amount of blood through cell reprogramming and ESM1 signaling could consequently improve our understanding of the molecular mechanisms involved in the process, and greatly increase the functionality and therapeutic potential of iPS‐ECs in the clinic. This can, in turn, prove tremendously important in the advancement of patient‐specific therapy, especially in regard to the treatment of EC dysfunction‐based vascular disease.

## Author Contributions

M.V.‐G. and S.K.: conception and design, collection and/or assembly of data, data analysis and interpretation, manuscript writing; C.M., R.C., D.C., M.E, A.C., D.D., M.T., K.O., E.P., C.Y., R.M.: collection and/or assembly of data; D.M.: provision of study material, final approval of manuscript; D.S.: collection and/or assembly of data, final approval of manuscript; A.Z., L.Z, D.G.: provision of study material, final approval of manuscript; N.L.: provision of study material, final approval of manuscript; A.W.S.: provision of study material, final approval of manuscript; A.M.: conception and design, collection and/or assembly of data, data analysis and interpretation, manuscript writing, financial support, final approval of manuscript.

## Disclosure of Potential Conflicts of Interest

The authors indicated no potential conflicts of interest.

## Supporting information


**Supplemental Experimental Procedures**

**Supplementary Figure S1** (A): Flow cytometry histogram plot showing that iPS cells are negative for the EC marker CD31. (B) Real‐time PCR showing how mRNA expression levels for the endothelial markers KDR, CD144 and eNOS change in iPS‐ECs during endothelial differentiation at day 9 before CD144 selection. (Data are means ±SEM [n = 3], *p < .05). (C) Flow cytometry histogram showing that the negative population after CD144 selection during iPS‐ECs differentiation is expressing the stromal marker CD90

**Supplementary Figure S2: Comparison of overall gene expression profiles for iPS cells vs iPS‐ECs:** (A) Scatter plot providing an overview of differential expression patterns between iPS cells and iPS‐ECs. Averaged normalized expression data were used to create this plot.
**(B)** Graph showing the fold change values and directions for the pluripotency‐associated genes Nanog, Oct4, Lin28 and Sox2 between iPS cells and iPS‐ECs. For all RNA sequencing analyses, n = 3.

**Supplementary Figure S3: iPS‐ECs display an endothelial transcriptional signature:** (A) Volcano Plot of differentially expressed genes in iPS cells versus iPS‐ECs depicting statistical significance as —log10(p‐values) on the y‐axis plotted against fold change as —log10(fold changes) on the x‐axis. (B) Graph depicting the top 10 most significantly enriched pathways of genes upregulated in iPS‐ECs compared to iPS cells. Results are displayed as —log10(p‐value). For all RNA sequencing analyses, n = 3.

**Supplementary Figure S4: Assessment of differential gene expression and enrichment patterns in iPS‐ECs vs. iPS cells:** Color heat map showing the results of gene functional classification, where this set of membrane proteins appeared as the most significantly enriched group (enrichment score = 5.705). Green shows an association between a gene and annotation term, while black indicates no association. [For functional annotation & enrichment analyses, an EASE score (modified Fisher's exact test p‐value) <0.1 defines significance. Genes with a fold change >30 & an FDR‐corrected p‐value of <0.05 were used for annotation in DAVID. For all RNA sequencing analyses, n = 3].

**Supplementary Figure S5: Gene ontology (GO) annotations for upregulated genes in iPS‐ECs vs. iPS cells:** (A) Graph displaying the top 10 most significantly enriched GO “Biological Process” terms annotated to genes upregulated in iPS‐ECs vs. iPS cells. Results are presented as —1og_10_(p‐value). (B) Graph displaying significantly enriched GO “Cellular Compartment” terms annotated to genes upregulated in iPS‐ECs vs. iPS cells. Results are presented as —1og_10_(p‐value). [For functional annotation & enrichment analyses, an EASE score (modified Fisher's exact test p‐value) <0.1 defines significance. Genes with a fold change >30 & an FDR‐corrected p‐value of <0.05 were used for annotation in DAVID. For all RNA sequencing analyses, n = 3].

**Supplementary Figure S6:** iPS‐ECs overexpressing ESM1 show upregulation of key proangiogenic markers and downregulation of antiangiogenic factors. The values were normalized so that the maximum overexpression (red) equalled 1 and the lowest downregulation (blue) equalled −1. No changes equal 0.

**Supplementary Figure S7:** ESM1 regulates EC marker expression from iPS cells in early stages of differentiation. Real Time PCR data showing comparison of ESM1 mRNA expression levels in iPS cells after transfected with ESM1 for 3 days. (Data are means ±SEM [n = 3], *p < .05, ***p < .001).

**Supplementary Figure S8: Comparison of overall gene expression profiles for iPS‐ECs (EX‐mCherry) vs. iPS‐ECs (EX‐ESM1)**:(A) Principal component analysis (PCA) for control iPSECs (EX‐mCherry) and iPS‐ECs overexpressing ESM1 (EX‐ESM1) replicates. Normalized expression values were used for PCA. (B) Volcano Plot of differentially expressed genes in iPSECs (EX‐mCherry) versus iPS‐ECs overexpressing ESM1 (EX‐ESM1) depicting statistical significance as —log10(p‐values) on the y‐axis plotted against fold change as —log10(fold changes).

**Supplementary Figure S9:** Real Time PCR comparing mRNA expression levels for ESM1, CX40 and the arterial marker Ephrin B2 between iPS‐ECs and human endothelial aortic cells (HAoECs). (Data are means ±SEM [n = 3], **p < .01).

**Supplementary Figure S10:** Immunofluorescent confocal image showing co‐staining of CX40 (red), eNOS (green) and DAPI (blue) in cells overexpressing eNOS‐GFP. Scale bars: 25 μm. (B) Real time is shown that the relative ESM1 mRNA expression levels are decreasing in late passages (after passage 15) of iPS‐ECs culture. (Data are means ±SEM [n = 3], **p < .01).
