# Supplementary material for: Enhanced Function of Induced Pluripotent Stem Cell‐Derived Endothelial Cells Through ESM1 Signaling
Source: Stem Cells. 2018 Nov 17;37(2):226–39. doi: 10.1002/stem.2936 (PMC6392130; doi:10.1002/stem.2936)
Supplement: Supplementary file 1 — Supplemental Experimental Procedures [file STEM-37-226-s001.pdf]

Supplemental Experimental Procedures

Methods &Materials

Cell culture media, serum, and cell culture supplements were purchased from ATCC, Merck Millipore, LONZA, Sigma, BD, Reprocell and Thermo Fisher Scientific. Human aortic endothelial cells (HAoECs) were bought from Brennan and co. via PromoCell, Ireland (C-12271). Magnetic beads were purchased from Miltenyi Biotec. Antibodies against ESM1 were purchased from abcam (ab103590) and R&D (AF1810) Antibodies against mCherry (ab125096), TRA-1-60 (ab16288), Lin28 (ab46020), and eNOS (ab76198), were purchased from Abcam. CD144 (VE-cadherin) (ab33168 & STJ96234) and Oct4 (ab19857 & STJ72238) were purchased from Abcam or St John's Laboratory. Connexin-40 (STJ96742) and NFKB1 (STJ99314) were purchased from St John's Laboratory. Antibodies against vWF (SC-8068) were purchased from Santa Cruz. VEGFR2 (MAB3571) and β-actin (MAB8929) were purchased from R&D. ZO-1 (40-2200) was purchased from Invitrogen. CD31/PECAM-1 (ab28364, ab76533, ab76553 & BBA7) was purchased from Abcam and R&D, while GAPDH (sc-25778 & ab8245, ab125247) was purchased from Santa Cruz and Abcam. The secondary antibodies for immunostaining anti-mouse Alexa 568, and anti-rabbit Alexa 488, anti-rabbit Alexa 568, anti-goat Alexa 568, anti-goat alexa 488 were purchased from Thermo Fisher Scientific. The secondary antibodies for Western blotting were purchased from Abcam (anti-goat ab6885) and BIO-RAD (anti-mouse #170-6516 and anti-rabbit #170-6515).

Blood mononuclear cells (MNCs) isolation and expansion

In this study 6 different were used. After obtaining written informed consent from each donor, 1 ml of non-mobilized peripheral blood were collected by venepuncture in EDTA-coated 4 ml tubes. The blood was gradient-separated by layering it on Histopaque solution (1:1 ratio) and spinning for 30 minutes at 550gx at room temperature (break OFF). The MNCs formed a buffy coat between the plasma layer and the Histopaque buffer layer and were collected using a soft plastic pipette. After 3 washes with PBS, the cells were resuspended in 1ml of MNC medium, which consisted of serum free medium/SFM* (Iscove's modified Dulbecco's medium (IMDM; 21056023, Invitrogen) was combined with Ham's F-12 Nutrient Mix (11765054, Invitrogen) with 0.5 ml of GlutaMAX™ Supplement (35050061, Life Technologies), 0.5 ml of Penicillin-Streptomycin-Glutamine (100x) (10378016, Life Technologies), 0.5 ml of ITS-X (51500-056, Invitrogen), 250 mg of BSA (A9418, Sigma), 2.5 mg of l-ascorbic acid (A8960, Sigma), 0.5 ml of chemically-defined lipid concentrate (11905031, Invitrogen) and 0.9 µl of 1-thioglycerol (98%; M6145, Sigma) (final concentration of 200 µM). SFM was supplemented with 100 ng/ml SCF (300-07, PeproTech), 2U/ml EPO (287-TC-500, R&D Systems), 10 ng/ml IL-3 (200-03, PeproTech), 1 µM dexamethasone (D2915, Sigma-Aldrich), 40 ng/ml IGF-1 (100-11, PeproTech) and 100 µg/ml holo-transferrin (2914-HT-100MG, R&D Systems). The cells were counted and plated at a density of ~4 million cells per ml. On day 3 and day 6, the medium was changed by collecting all cells and spinning for 5 minutes at 250gx. The cells were cryopreserved from day 7 in freezing medium (50% FBS, 40% SFM and 10% DMSO) or used for reprogramming immediately.

Cell reprogramming

2 million cells were transfected with 10 µg of plasmid DNA: 8 µg of pEB-C5 (overexpressing Oct4, Sox2, Klf4, c-Myc and Lin28) and 2 µg of pEB-Tg (overexpressing SV40 large T antigen) using the Lonza CD34 nucleofector kit and Amaxa nucleofector (program T-016). After electroporation, the cells were plated in 2 ml of MNC medium in a well of a 12-well plate. On day 2 after transfection, the cells were collected, counted and seeded onto inactivated MEFs in reprogramming medium at a density of 100000 – 80000 cells per well. Reprogramming medium consisted of KnockOut™ DMEM/F-12 (12660012, Invitrogen) supplemented with GlutaMAX™ Supplement (35050061, Life Technologies), Penicillin-Streptomycin-Glutamine-100x (10378016, Life Technologies) and MEM NEAA (11140050) 1:100 to make basal medium. Basal medium was supplemented with 20% KnockOut™ Serum Replacement and 10ng/ml FGF2. NaB was added to the medium at a concentration of 0.25 mM until colonies were picked. Reprogramming medium (with NaB) was changed every day. 9 days after transfection, the reprogramming medium was replaced with conditioned medium containing FGF2 (10 ng/ml) and NaB (0.25 mM). The medium was changed every day. Colonies appeared from day 9. Once the colonies were picked, cell lines were established and cultured in reprogramming medium supplemented with FGF2.

Human iPS-Cells differentiation

Human induced pluripotent stem (iPS) cells cultured under feeder-free conditions were detached using dissociation solution (Reprocell) and seeded on mouse collagen IV (Cultrex Mouse Collagen IV (3410-010-01, R&D) in EGM-2 media (Lonza) 10% FBS. The medium was supplemented with 25 ng/ml BMP4, 12 ng/ml Activin A, 8 µM CHIR99021 and 20 ng/ml FGF2 (MACS). After 48 hours (day 2 of differentiation), the medium was replaced with EGM-2 10% FBS supplemented with 200 ng/ml VEGF (Life Technologies), 10 ng/ml FGF2 and 10 µM LY364947 (Sigma) and was refreshed every other day. On day 6 of differentiation, MACS-mediated magnetic selection for CD144-expressing cells was performed using MicroBeads Kit (Miltenyi Biotec), as we have previously shown (Cochrane et al., 2017). The positively selected cells were seeded on mouse collagen IV-coated plates in EGM-2 10% FBS media supplemented with 50 ng/ml VEGF and 10 µM LY364947. ESM1 was overexpressed or knocked down by lentiviral gene transfer on day 3 after CD144 selection and the cells were harvested after 48 or 72 hours, respectively, for further analysis or for use in the *in vivo* experiments.

RNA extraction, reverse transcriptase-polymerase chain reaction (RT-PCR) and real-time PCR

Cells were harvested and washed with cold PBS. RNA was extracted and purified using the RNeasy mini kit (QIAGEN) according to the manufacturer's instructions. RT-PCR and real time PCR were performed as previously described. (Cochrane et al., 2017; Margariti et al., 2012; Margariti et al., 2010) RNA yield was determined using the NanoDrop spectrophotometer (NanoDrop Technologies). Total RNA (2 µg) was converted to 2 µg of cDNA (relative to RNA amount) for each sample using High-Capacity cDNA Reverse Transcription Kit with RNase Inhibitor (#4374966 Thermo Fisher Scientific). Quantitative PCR (qPCR) was performed using Power SYBR® Green PCR Master Mix in a 10 µl reaction (Life Technologies) and *Ct* values were measured using a LightCycler 480 sequence detector (Roche). GAPDH or RPL12 served as the endogenous controls to normalize the amounts of RNA in each sample. For each sample, PCR was performed in duplicate in a 384-well reaction plate (LightCycler 480 Roche real time PCR plates). The gene was considered undetectable beyond 35 cycles. The primer set sequences are shown in Table 1 below:

forward: 5'-AGAACATGTGTAAGCTGCGG-3' and reverse: 5'-GTTGCCTCTCACTCGGTTC-3′ for Oct4,
forward: 5'-GCAGAAGCGCAGATCAAAAG-3' and reverse: 5'-CGGACATGAGGCTACCATATG-3′ for Lin28
forward: 5'-GAAATACCTCAGCCTCCAGC-3' and reverse: 5'-GCGTCACACCATTGCTATTC-3′ for Nanog
forward: 5'-ATAGAAGGTGCCCAGGAAAAG-3' and reverse: 5'-GTCTTCAGTTCCCCTCCATTG-3′ for KDR
forward: 5'-AAACACCTCACTTCCCCATC-3' and reverse: 5'-ACCTTGCCCACATATTCTCC-3′ for CD144,
forward: 5'-GGTACATGAGCACTGAGATCG-3' and reverse: 5'-GCCACGTTGATTTCCACTG-3′ for eNOS,
forward: 5'-GCACGGCTTTTGTTCAGATG-3' and reverse: 5'-CGGTTGAAGGTGAGACTGG-3′ for TERT1,
forward: 5'-ATGGACGCCGAACTCATC-3' and reverse: 5'-CCAAGTCCGAGAAGCAGTC-3′ for Cbp/300 CITED4
forward: 5'-TCATGGTGTGGGCAAAGG-3' and reverse: 5'-CGTTCAGCTCCTTCCACG-3′ for Sox18
forward: 5'-AGAATCCAGACCTGCACAAC-3' and reverse: 5'-GCCGGTACTTGTAGTTGGG-3′ for Sox17,
forward: 5'-GAGAAGCCCCAGACCAAAC-3' and reverse: 5'-ACCACACCTGACACCTTTTC-3′ for TCF3
forward: 5'-AGTAGCTGTCAAATGGCCTTC-3' and reverse: 5'-TTAGTTCGGCTTTGAGGGTG-3′ for FOXC1
forward: 5'-CAAGAACTCGCTGATCCAAATG-3' and reverse: 5'-GCTGTACGTTCCTTCTCCTTC-3′ for mTOR
forward: 5'-GGCAAGACCTACATCTCCAAG-3' and reverse: 5'-ATGGCTTCCTCATTGTCGG-3′ for PFKFβ3
forward: 5'-CATCAACCGCAACGAGGA-3' and reverse: 5'-GGTCATGGGTCACGTCAG-3′ for GLUT1
forward: 5'-ATGGCTGACACATTCCTGG-3' and reverse 5'-CATCTCCTTCAACGTCTCCAC-3′ for PKM
forward: 5'-CCCTCAAAGTAAGACCAGTAGC-3' and reverse: 5'-CACAGTCTCCAAGAAGCTCTAC-3′ for SIRT1
forward: 5'-CCGCTGGAGACACAATCATATC-3' and reverse: 5'-ACTTCCTCAAGTTGCTGGTC-3′ for HIF1α
forward: 5'-CCCATGTCTCCACCTTCAAG-3' and reverse: 5'-GGCTTGCTCTTCATACTCCAG-3′ for EPAS1
forward: 5'-GGTCTATCTTAATCTGGTGCTGG-3' and reverse: 5'-TGGATATAGGCTAAACTTCGGAAC-3′ for GSK3β
forward: 5'-CTACATGCTAGACCTGTATCGC-3' and reverse: 5'-CCCACTCGTTTCTGGTAGTTC-3′ for BMP2
forward: 5'-GCCCTCACAAACACCTACAG-3' and reverse: 5'-TCATAACTCCGCCCATTCAC-3′ for PAX6
forward: 5'-GGAAGCCTAACTACAGCGAG-3' and reverse: 5'-CAGAGTCCCAGATGAGCATTG-3′ for SNAI1
forward: 5'-AGAAAGGCTGGGCGAAGAC-3' and reverse: 5'-TAGCACGTGGTCAGATGCAG-3′ for CD34
forward: 5'-AACCTCTTTGCCCTGTATGAC-3' and reverse: 5'-CTGCTCATTGTCGTTGGTTC-3′ for JAK1
forward: 5'-TGCCTGGACAAGATCAATGAG-3' and reverse: 5'-CAGGTGTAAGTGTTGGGTCC-3′ for NOTCH1
forward: 5'-TTCTGGGCACAAACACAAAAG-3' and reverse: 5'-TCAGTCACAATCAGGGAAGC-3′ for STAT3
forward: 5'-CTCAGCTACGCCTTCTCG-3' and reverse: 5'-ACTGTCCATTTTCTCCTTCTCTG-3′ for TWIST1
forward: 5'-ACAAGCCATGACTCAGGATG-3' and reverse: 5'-TGTTTCACTGGAGCACTCTG-3′ for YAP1
forward: 5'-CCAGGTTGCAAGATTTAATGACC-3' and reverse: 5'-TTTTGATGGCTCTGTGGTAGG-3′ for RUNX1
forward: 5'-GCGGGCTCTATCACAAAATG-3' and reverse: 5'-TCCCCATTGGCATTCCTC-3′ for GATA3
forward: 5'-CACAACGCCGAGCTCAG-3' and reverse: 5'-GGCCGGTACTTGTAGTTGG-3′ for Sox7
forward: 5'-CTGGGTTTATGGGAAGGACG-3' and reverse: 5'-CAGGGAGACAGAACACATAAGAC-3′ for TEK

forward: 5'-CGAGTACATCTTCAAGCCATCC-3' and reverse: 5'-TGGTGAGGTTTGATCCGC-3′ for VEGF-A
forward: 5'-CCTACACCAAGAGTTCGCATC-3' and reverse: 5'-TGTGCTTTCGGTAGTGGC-3′ for Klf2
forward: 5'-TGGTACCCAGTGCTTTTGAG-3' and reverse: 5'-CTCCGATAGTCCATAGCAAGG-3′ for HEY1
forward: 5'-CTGTTCCTCACCATCCCTTTC-3' and reverse: 5'-CAAGGAGATGAGAAACGAGGTG-3′ for MALL
forward: 5'-GGTGTCAGCCTTCTAATGGG-3' and reverse: 5'-TCAGGCATTTTCCCGTCC-3′ for ESM1
forward: 5'-TCAGCAACTCATTCCCACAG-3' and reverse: 5'-GCAGGGCTGGTTTAGGATAG-3′ for WARS
forward: 5'-GGACTATGAGGGCAAGAACTG-3' and reverse: 5'-AAATATACCGCACCCCTTCAG-3′ for JAG1
forward: 5'-AGACAAGAGCCATGAAGATCC-3' and reverse: 5'-ACTTCTCCCATTTGTACCAGC-3′ for Ephrin B2

Table 1: Primers used for Real Time PCR

RNA Sequencing

Cells were briefly washed with PBS and harvested using QIAzol lysis buffer. Total mRNA and miRNA was extracted and purified using miRNAeasy (QIAGEN). The RNA concentration was determined using NanoDrop spectrophotometer and the integrity of the sample was assessed using Agilent RNA 6000 Nano Kit and Agilent 2100 Bioanalyzer (Agilent Technologies). RNA sequencing libraries were prepared using a stranded KAPPA RNA-Seq Kit with RiboErase (KapaBiosystems) according to the manufacturer's instructions with 1 µg total RNA in 10 µL RNase-free water as an input. This protocol included a step to deplete ribosomal RNA (rRNA) by hybridization with complementary DNA (cDNA) oligonucleotides. Treatment with RNase H removed rRNA duplexed to DNA. DNase treatment removed hybridization oligonucleotides from the sample prior to cDNA synthesis. rRNA-depleted RNA was eluted and fragmented to the desired size by incubating at a high temperature in the presence of Mg^{2+}. 1st strand cDNA synthesis was performed using random primers. This was followed by 2nd strand cDNA synthesis to convert the cDNA/RNA hybrid to double-stranded (ds) cDNA, while the 2nd strand was marked with dUTP. A-tail (dAMP) addition to the 3'-end of dscDNA fragments was achieved by resuspending beads in A-Tailing Buffer-1x and storing at 4°C for ≤24 hours. An adapter ligation step followed, where 3'-dTMP adapters were ligated to 3'-dAMP library fragments. Lastly, library amplification involved adapter-ligated library DNA being amplified by PCR. RNA sequencing libraries were quantified using qPCR with the KAPA Library Quantification Kit (KapaBiosystems) (20µL reaction volume) containing KAPA SYBRFAST DNA Polymerase. This was followed by normalization, pooling and sequencing on a NextSeq 500 (Illumina). FastaQ files were aligned using the CLC Genomics Workbench 10.0.1 (Qiagen, https://www.qiagenbioinformatics.com). Sequencing reads were mapped to the human reference genome (hg38), allowing up to 2 mismatches and up to 10 hits per read. Reference sequences were annotated with genes and transcripts. Reference content was mapped to genes only. Expression values per gene or transcript were defined by total counts.To ensure sample comparability and that assumptions on the data were met for further analyses, expression values were transformed (log_{10}) and normalized by total read counts (reads per 1,000,000). Normalized expression values were used for further high-level bioinformatic analyses. Data quality control steps were undertaken using CLC. To view overall distributions at the group-level, box plots were created. Hierarchical clustering (Manhattan distance; single linkage) of samples was used to assess the relative similarity of group expression profiles. To assess the direction of variability in the datasets, principal component analysis of normalized expression values was performed. Statistical analysis was undertaken using the 'Empirical Analysis of Differential Gene Expression' option implemented in CLC. This makes use of the 'Exact Test' methods that were developed by Robinson and Smyth (Robinson & Smyth, 2008) and incorporated into the EdgeR Bioconductor package (Robinson et al., 2010). For statistical analysis, original expression values were used, as this test automatically normalizes data. For comparative expression analyses, fold change values were positive when the mean expression value in group two was positive relative to that in group one and vice versa. For RNA sequencing analyses, a fold change of $\geq$2 identified differential expression. To take adjust for multiple testing (Dudoit et al., 2003), false-discovery rate (FDR)-adjusted p-values were calculated using the Benjamini-Hochberg method (Benjamini and Hochberg, 1995). An FDR-adjusted p-value of $\leq$0.05 was chosen to indicate statistical significance. Comparative gene expression data was filtered according to the defined fold change and FDR-adjusted p-value thresholds. Statistical analysis results were displayed as volcano plots, where $-log_{10}$(p-values) are plotted against log_2(fold change) for each feature. Hierarchical clustering of features (Manhattan distance; single linkage) was used to identify genes with similar expression patterns over the samples. K-mean clustering was also performed (number of partitions=5; distance metric=Manhattan distance). For all RNA sequencing steps implemented in CLC, default settings were used unless stated otherwise.

Functional annotation and gene enrichment
Functional annotation and gene enrichment analyses of RNA sequencing expression data were undertaken using the DAVID bioinformatic database version 6.7 (Huang da et al., 2009; Huang da et al., 2008). Functional annotation was undertaken for genes that were upregulated in 1. iPS-ECs vs. iPS cells and 2. HUVECs vs. iPS-ECs. Genes with a fold change $\geq$30 were chosen for functional annotation and enrichment analyses. Functional annotation was undertaken for the 'Biological Process', 'Cellular Component' and 'Molecular Function' gene ontology (GO) categories, as well as, 'UP_tissue expression'. Pathway analysis for BBID (Biological Biochemical Image Database), Biocarta, KEGG (Kyoto Encyclopedia of Genes and Genomes) and Panther annotations was undertaken. The 'functional annotation chart' option was implemented for these analyses. This option provides an annotation term-focused view that lists annotations and their associated genes under study. For functional annotations using DAVID, a maximum EASE score threshold was set at 0.1. The EASE score is a modified and more conservative 'Fisher's Exact' p-value for gene enrichment analyses utilized by DAVID. The EASE score ranges from 0-1, where 0 indicates perfect enrichment. Upregulated genes were also grouped according to function using the 'Gene Functional Annotation' option implemented in DAVID. This gene-centric modular analysis classifies upregulated genes into groups before ranking group importance based on enrichment scores. The enrichment score is calculated from the geometric mean of the EASE score for all terms involved in a given gene group. For this analysis, classification stringency was set to medium. Kappa similarity and classification thresholds were used at the default levels defined by DAVID.

Immunofluorescence staining
The procedure for immunofluorescent staining was similar to that described previously (Cochrane et al., 2017; Margariti et al., 2012; Margariti et al., 2010). Briefly, cells were fixed with 4% paraformaldehyde or ice cold methanol for 10 min, permeabilized with 0.1% Triton X-100 in PBS for 5 min and blocked in 5% goat or donkey serum in PBS for 30 min at room temperature. Next, cells were incubated with primary antibodies for 1 hour at 37 ºC. After three 5-minute washes with PBS, the specimens were incubated with fluorochrome conjugated secondary antibody for 45 minutes at 37 ºC. The cells were counterstained with 4'-6-diamino-2-phenylindole (DAPI), mounted and examined under a fluorescence microscope (OLYMPUS IX51) or confocal microscope (Nikon Eclipse Ti).

Immunoblotting
The method used was similar to that described previously (Margariti et al., 2010). Cells were harvested and washed with cold PBS, re-suspended in RIPA buffer (SIGMA) supplemented with protease inhibitors (Roche) and sonicated (two times of 6 seconds each) using Soniprep 150 (MSE). The protein concentration was determined using the Quick Start™ Bradford Protein Assay (500-0205, BIO-RAD). 50 µg of whole lysate was applied to SDS-PAGE and transferred to Hybond PVDF membrane (GE Health), followed by standard immunoblotting procedure. The bound primary antibodies were detected using horseradish peroxidase (HRP)-conjugated secondary antibody and Clarity Western ECL Substrate (170-5060, BIO-RAD).

Co-immunoprecipitation (Co-IP)
To perform Co-IP, antibody against the target proteins (eNOS-GFP or CX40) was coupled to Protein A/G agarose beads (Santa Cruz, sc2003), and the complexes containing the target protein were immunoprecipitated. Protein components in the complexes were visualized by immunoblotting, as described above.

Human Magnetic Luminex Assay
The concentration of ESM1 released into the cell culture media of iPS-ECs (1 million cells per condition) was detected by Human Magnetic Luminex Assay (R&D; LXSAHM) according to the manufacturer's protocol.

Plasmid Generation
Plasmids for EX-mCherry control (pReceiver-Lv224), and EX-ESM1 containing the ESM1 gene (NM_007036.4), were purchased from Genecopoia. pEB-C5 was a gift from Linzhao Cheng (Addgene plasmid # 28213). pEB-Tg was a gift from Linzhao Cheng (Addgene plasmid # 28220) (Chou et al., 2011). Plasmids were amplified by incubating in Competent Cells (Promega #JM109) for 30 minutes on ice. After a heat shock step at 42°C for 90 seconds, samples were spun for 2 minutes at 4000 rpm. 200 µl of the supernatant were then used to resuspend pellets and spread on agar plates supplemented with ampicillin. The plates were cultured at 37ºC overnight. Next day, colonies were

picked and amplified in 5 ml of LB broth with ampicillin by incubating them overnight at 37ºC and shaking at 200 rpm. The following day, the amplified cells were pelleted by centrifugation and the plasmids were purified using with QIAprep Spin Miniprep Kit (QIAGEN) according to manufacturer's instructions.

Transfection

iPS-ECs were infected or transfected with the ESM1 plasmid or empty vectors according to manufacturer's protocol, and cultured in EGM-2 media for three additional days. The differentiated cells were harvested on day 6 and endothelial marker expression was tested at both transcriptional and protein level. The efficiency of the transfection/infection was 60-70%.

Flow cytometry analysis

iPS-ECs were detached using TrypLE Express Enzyme (Life Technologies) and collected. The cells were spun, resuspended in PBS with 10% FBS and incubated for 10 minutes at 4ºC for blocking. Next, the cells were labelled with either anti-IgG, anti-CD144 antibodies conjugated with APC, anti-CD31 conjugated with PE, anti-CD90 with FITC (Thy-1) Monoclonal Antibody (eBiosciences) by incubating them for 30 minutes at 4ºC. The cells were then washed and resuspended in PBS 10% FBS and analysed with the Attune NxT Flow Cytometre (Thermo Fisher Scientific). The analysis of the data was performed using FlowJo software.

Ac-LDL uptake assay

To detect acetylated low-density lipoprotein (LDL) uptake by iPS-ECs, cells were incubated with Dil-ac-LDL (Molecular Probes) for 4 hours and were examined and photographed under a fluorescent microscope.

***In vitro* tube formation assay**

24-well plates were coated with 289 µl/well of Matrigel Matrix (Corning). The plates were incubated at 37ºC for 30 minutes. 1.2×10^5 cells were plated in each well at a concentration of 4×10^5 cells/ml. The cells were incubated for up to 18 hours at 37ºC 5% CO_2. Staining of the tubes was performed as described in the Immunofluorescence staining section above.

Lentiviral particle transduction

Lentiviral particles were produced using MISSION shESM1 or shCX40 plasmids (Sigma-Aldrich) according to the protocol provided and as previously described (Cochrane et al., 2017; Margariti et al., 2012; Margariti et al., 2010). The shRNA Non-Targeting vector was used as a negative control. Briefly, 293-T cells were transfected with the lentiviral vector and the packaging plasmids, pCMV-dR8.2 and pCMV-VSV-G (both obtained from Addgene) using Fugene 6 or Endofectin Lenti. The supernatant containing the lentivirus was harvested 48h later, filtered, aliquoted and stored at -80°C. p24 antigen ELISA (Zeptometrix) was used to determine the viral titre. The Transducing Unit (TU) was calculated using the conversion factor recommended by the manufacturer (10^4 physical particles per pg of p24 and 1 transducing unit per 10^3 physical particles for a VSV-G pseudotyped lentiviral vector), with 1 pg of p24 antigen converted to 10 Transducing Units (TU). For the lentiviral particle transduction for the constructs purchased from Genecopoeia, a similar approach was used following the manufacturer's instructions. For lentiviral infection, the cells were incubated with shESM1 or non-targeting control (1×10^7TU/ml) in complete medium supplemented with 10 µg/ml of Polybrene for 24 hours. After that time, fresh medium was added to the cells. The plates were harvested at 72 hours for further analysis. The efficiency of the infection was 70-80%.

Luciferase Assay

For the luciferase reporter assays, human iPS-ECs were seeded into 12-well plates and treated with 5 µM of NKFB inhibitor (BAY 11-7082, Cayman Chemical) or DMSO control. Cells were co-transfected with EX-ESM1, Renilla-TK control and the PGl3-Luc CX40 reporter (#VB171020-1028vjf, VectorBuilder). Briefly, 0.33 µg/well of the reporter plasmid was co-transfected with EX-ESM1 or EX-mCherry (0.17 µg/well) using Endofectin Max according to the manufacturer's protocol. pGL3-Luc Renilla (0.1µg/well) was included in all transfection assays as an internal control. Luciferase and Renilla (Promega) activity assays were detected 48 hours after transfection using the manufacturer's protocol. Relative luciferase units (RLU) were defined as the ratio of Luciferase activity to Renilla activity with that of control set as 1.0.

In vivo teratoma assay
$1x10^6$ iPS cells were suspended in Matrigel and injected subcutaneously in mice homozygous for the severe combined immune deficiency spontaneous mutation Prkdcscid SCID. 10 weeks later, the Matrigel plugs were harvested, fixed in 4% PFA overnight and embedded in paraffin. 5 µm sections were cut and mounted on Superfrost Plus microscope slides (ThermoFisher J1800AMNZ). Deparaffinization and rehydration of the mounted sections were followed by standard H&E staining procedures or heat-induced antigen retrieval using Sodium citrate buffer (10 mM Sodium citrate, 0.05% Tween 20, pH 6.0) followed by immunohistochemistry.

In vivo tube formation assay
$1x10^6$ iPS-ECs overexpressing an mCherry control plasmid (EX-mCherry) or ESM1-mCherry plasmid (EX-ESM1) were mixed with 200 µl of Matrigel Matrix (Growth factor reduced, Corning) and subcutaneously injected in NOD.CB17-Prkdcscid/NcrCrl mice. A total of three injections were conducted for each group. Seven days later the mice were sacrificed and the plugs were harvested, fixed in 4% PFA overnight and embedded in paraffin. 5µm sections were cut, mounted and fixed. Specimens were then placed in a humidified chamber and blocked in 5% donkey serum in PBS for 30 minutes at 37˚C and incubated with primary antibodies rabbit anti-CD144 overnight at 4ºC. The bound primary antibodies were revealed by incubation with the secondary antibody; anti-rabbit Alexa488, at 37^oC for 45 minutes. Specimens were counterstained with 4',6-diamidino-2-phenylindole (DAPI; Sigma-Aldrich), mounted in Floromount-G (Cytomation; DAKO, Glostrup, Denmark), and examined with a fluorescence microscope (Axioplan 2 imaging; Zeiss) or SP5 confocal microscope (Leica, Germany). Immunostaining was assessed and capillary density was calculated as capillary number/mm^2.

Experimental hindlimb ischemia
Unilateral hind limb ischaemia was induced in 10-week-old NOD/SCID mice by ligation of the left femoral artery. 2% isoflurane at 1L/min O_2 was used to induce anaesthesia and animals were maintained throughout surgery at 1.5% isoflurane on a 37^oC heat pad. 200 µl Vetergesic was given prior to surgery commencing as pain relief. After ligation with nylon sutures, mice were injected intramuscularly with PBS vehicle control or $1x10^6$ iPS-ECs transfected with EX-mCherry or EX-ESM1. 50 µl of PBS or PBS-cell suspension was administered along the adductor muscle adjacent to site of ligation. Site of wound was then closed using 5.0 vicryl sutures (Ethicon, USA). Limb reperfusion was measured in comparison to untreated limb at 7 and 14 days after surgery by Laser Doppler Imaging (Moor Instruments, UK). At 14 days, the mice were sacrificed and adductor and gastrocnemius muscles harvested. Tissue was fixed overnight in 4% paraformaldehyde/PBS solution, followed by sucrose cryoprotection before embedding in OCT medium. In short, 5-12 µm sections were cut from cryopreserved OCT blocks or paraffin blocks and mounted on Superfrost Plus microscope slides (ThermoFisher). Antigen retrieval was performed by boiling the specimens in Sodium Citrate buffer (10 mM Sodium citrate, 0.05% Tween 20, pH 6.0) for 20 minutes. Specimens were washed twice with TBS with 0.025% Triton X-100. In a humidified chamber, specimens were blocked with 10% donkey serum with 1% BSA in TBS for 2h at room temperature. The primary antibodies were diluted in TBS with 1% BSA and applied on the specimens followed by incubation overnight at 4ºC. Next day, the slides were washed 3 times with TBS with 0.025% Triton X-100 and then incubated for 1 hour at room temperature with secondary antibodies diluted in TBS with 1% BSA. The specimens were washed 2 times with TBS and counterstained with with 4'-6-diamino-2-phenylindole (DAPI). Vectashield mounting medium was used to mount coverslips on the specimens.

Generation of stable transfectants over-expressing GFP-tagged eNOS
Human Embryonic Kidney cells (HEK 293A Clontech) were transfected with a plasmid (pcDNA3) encoding an eNOS-GFP expression construct which contains eNOS fused in frame with GFP at the C-terminus to allow generation of an eNOS-GFP fusion protein (McDonald et al., 2004). Stable transfectants were selected by culturing in the presence of 300µg/ml G418 (Sigma, UK) for 2-3 weeks after which time untransfected cells had died and resistant colonies had remained. Individual G418 resistant colonies were selected by fluorescence microscopy and single clones selected using cloning rings. Expression of eNOS-GFP (160 kDa) was confirmed by western blotting and normal localization of eNOS to the plasma membrane and Golgi complex verified by confocal microscopy.

Angiogenesis array
iPS-ECs were transfected with either EX-mCherry or EX-ESM1 using Endofectin Max as described before. 72 hours after transfection, the media were harvested and spun at 1000 rpm. The

supernatants were collected and either used straight away or frozen in aliquots. 3 repeats of each condition were pulled together in one membrane for the arrays. The angiogenesis arrays were performed using the Proteome Profiler Human Angiogenesis Array Kit (ARY007, Bio-Techne Ltd.) according to the manufacturer's instructions.The blots were imaged using SYNGENE G:BOX XX6 and analysed with HLImage++ (Western Vision). The heat map was generated using R-Studio.

Statistical Analysis
Data are expressed as mean ± SEM and were analyzed using GraphPad Prism 5 software with a two-tailed Student's *t* test for two groups or pairwise comparisons and Bonferroni post test (to one way ANOVA). Values were considered significant when $p<0.05$, ** $p<0.01$, ***$p<0.001$.

References
Chou, B.K., Mali, P., Huang, X., Ye, Z., Dowey, S.N., Resar, L.M., Zou, C., Zhang, Y.A., Tong, J., and Cheng, L. (2011). Efficient human iPS cell derivation by a non-integrating plasmid from blood cells with unique epigenetic and gene expression signatures. Cell Res *21*, 518-529.
Cochrane, A., Kelaini, S., Tsifaki, M., Bojdo, J., Vila-Gonzalez, M., Drehmer, D., Caines, R., Magee, C., Eleftheriadou, M., Hu, Y.*, et al.* (2017). Quaking Is a Key Regulator of Endothelial Cell Differentiation, Neovascularization, and Angiogenesis. Stem cells *35*, 952-966.
Huang da, W., Sherman, B.T., and Lempicki, R.A. (2009). Systematic and integrative analysis of large gene lists using DAVID bioinformatics resources. Nature protocols *4*, 44-57.
Huang da, W., Sherman, B.T., Stephens, R., Baseler, M.W., Lane, H.C., and Lempicki, R.A. (2008). DAVID gene ID conversion tool. Bioinformation *2*, 428-430.
Margariti, A., Winkler, B., Karamariti, E., Zampetaki, A., Tsai, T.N., Baban, D., Ragoussis, J., Huang, Y., Han, J.D., Zeng, L.*, et al.* (2012). Direct reprogramming of fibroblasts into endothelial cells capable of angiogenesis and reendothelialization in tissue-engineered vessels. Proceedings of the National Academy of Sciences of the United States of America *109*, 13793-13798.
Margariti, A., Zampetaki, A., Xiao, Q., Zhou, B., Karamariti, E., Martin, D., Yin, X., Mayr, M., Li, H., Zhang, Z.*, et al.* (2010). Histone deacetylase 7 controls endothelial cell growth through modulation of beta-catenin. Circulation research *106*, 1202-1211.
McDonald, D.M., Alp, N.J., and Channon, K.M. (2004). Functional comparison of the endothelial nitric oxide synthase Glu298Asp polymorphic variants in human endothelial cells. Pharmacogenetics *14*, 831-839.
