# Supplementary figures and images for: Enhanced Function of Induced Pluripotent Stem Cell‐Derived Endothelial Cells Through ESM1 Signaling
Source: Stem Cells. 2018 Nov 17;37(2):226–39. doi: 10.1002/stem.2936 (PMC6392130; doi:10.1002/stem.2936)

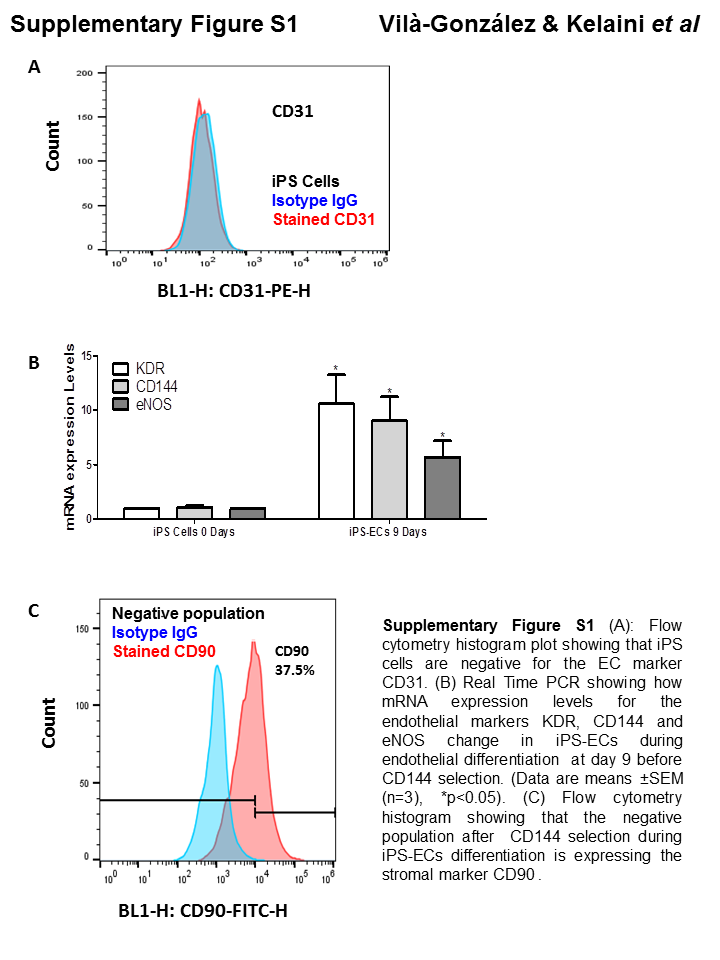

Supplement: Supplementary file 2 — Supplementary Figure S1 (A): Flow cytometry histogram plot showing that iPS cells are negative for the EC marker CD31. (B) Real‐time PCR showing how mRNA expression levels for the endothelial markers KDR, CD144 and eNOS change in iPS‐ECs during endothelial differentiation at day 9 before CD144 selection. (Data are means ±SEM [n = 3], *p < .05). (C) Flow cytometry histogram showing that the negative population after CD144 selection during iPS‐ECs differentiation is expressing the stromal marker CD90 [file STEM-37-226-s002.TIF]

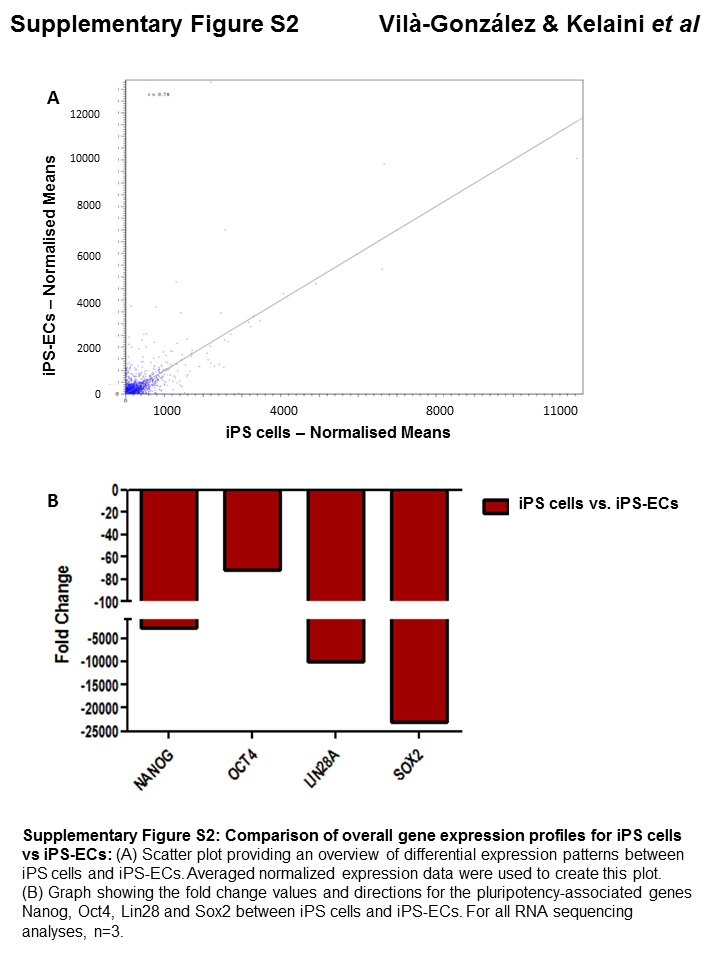

Supplement: Supplementary file 3 — Supplementary Figure S2: Comparison of overall gene expression profiles for iPS cells vs iPS‐ECs: (A) Scatter plot providing an overview of differential expression patterns between iPS cells and iPS‐ECs. Averaged normalized expression data were used to create this plot. (B) Graph showing the fold change values and directions for the pluripotency‐associated genes Nanog, Oct4, Lin28 and Sox2 between iPS cells and iPS‐ECs. For all RNA sequencing analyses, n = 3. [file STEM-37-226-s003.TIF]

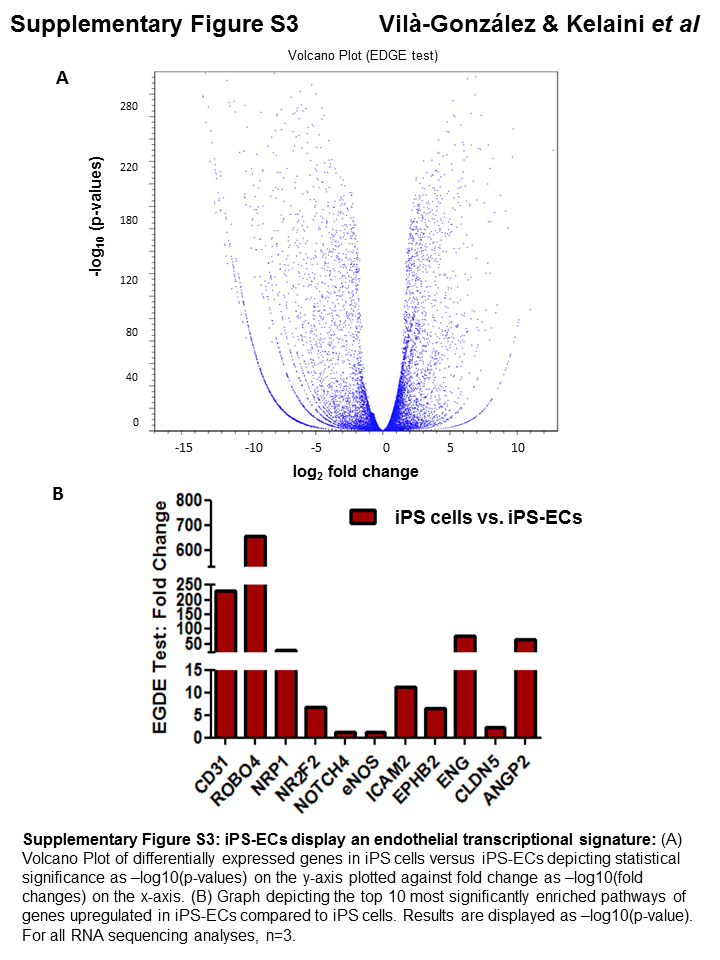

Supplement: Supplementary file 4 — Supplementary Figure S3: iPS‐ECs display an endothelial transcriptional signature: (A) Volcano Plot of differentially expressed genes in iPS cells versus iPS‐ECs depicting statistical significance as —log10(p‐values) on the y‐axis plotted against fold change as —log10(fold changes) on the x‐axis. (B) Graph depicting the top 10 most significantly enriched pathways of genes upregulated in iPS‐ECs compared to iPS cells. Results are displayed as —log10(p‐value). For all RNA sequencing analyses, n = 3. [file STEM-37-226-s004.TIF]

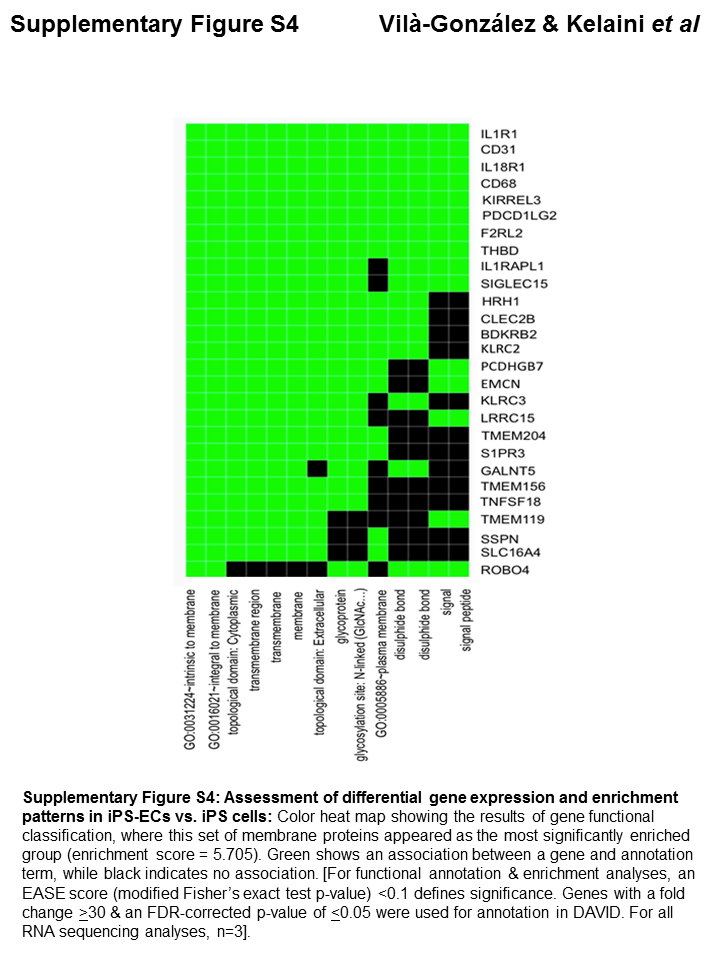

Supplement: Supplementary file 5 — Supplementary Figure S4: Assessment of differential gene expression and enrichment patterns in iPS‐ECs vs. iPS cells: Color heat map showing the results of gene functional classification, where this set of membrane proteins appeared as the most significantly enriched group (enrichment score = 5.705). Green shows an association between a gene and annotation term, while black indicates no association. [For functional annotation & enrichment analyses, an EASE score (modified Fisher's exact test p‐value) <0.1 defines significance. Genes with a fold change >30 & an FDR‐corrected p‐value of <0.05 were used for annotation in DAVID. For all RNA sequencing analyses, n = 3]. [file STEM-37-226-s005.TIF]

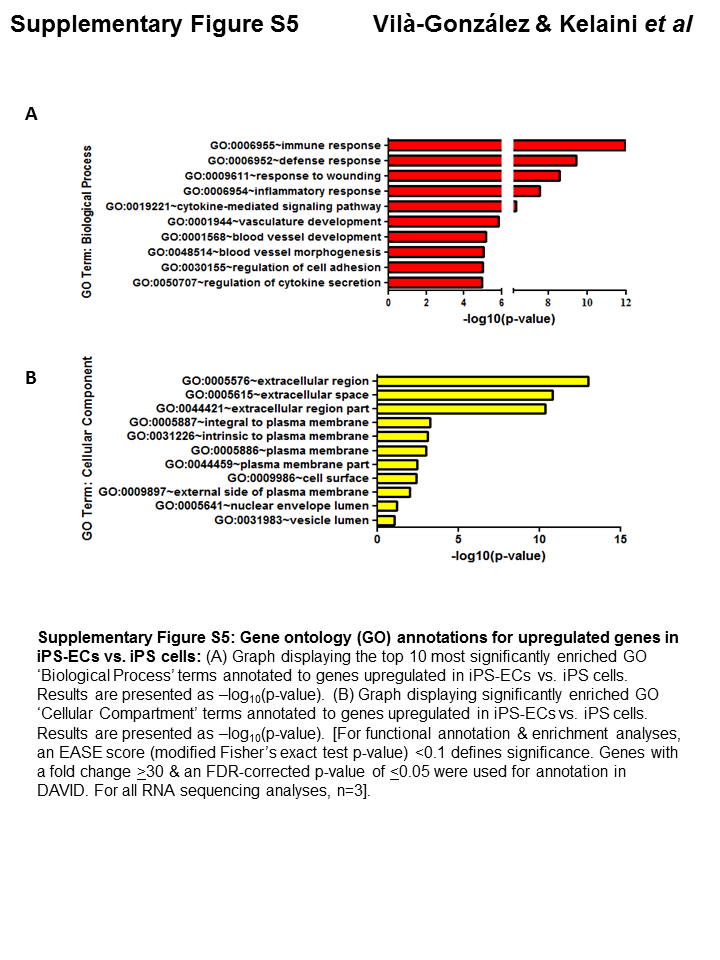

Supplement: Supplementary file 6 — Supplementary Figure S5: Gene ontology (GO) annotations for upregulated genes in iPS‐ECs vs. iPS cells: (A) Graph displaying the top 10 most significantly enriched GO “Biological Process” terms annotated to genes upregulated in iPS‐ECs vs. iPS cells. Results are presented as —1og10(p‐value). (B) Graph displaying significantly enriched GO “Cellular Compartment” terms annotated to genes upregulated in iPS‐ECs vs. iPS cells. Results are presented as —1og10(p‐value). [For functional annotation & enrichment analyses, an EASE score (modified Fisher's exact test p‐value) <0.1 defines significance. Genes with a fold change >30 & an FDR‐corrected p‐value of <0.05 were used for annotation in DAVID. For all RNA sequencing analyses, n = 3]. [file STEM-37-226-s006.TIF]

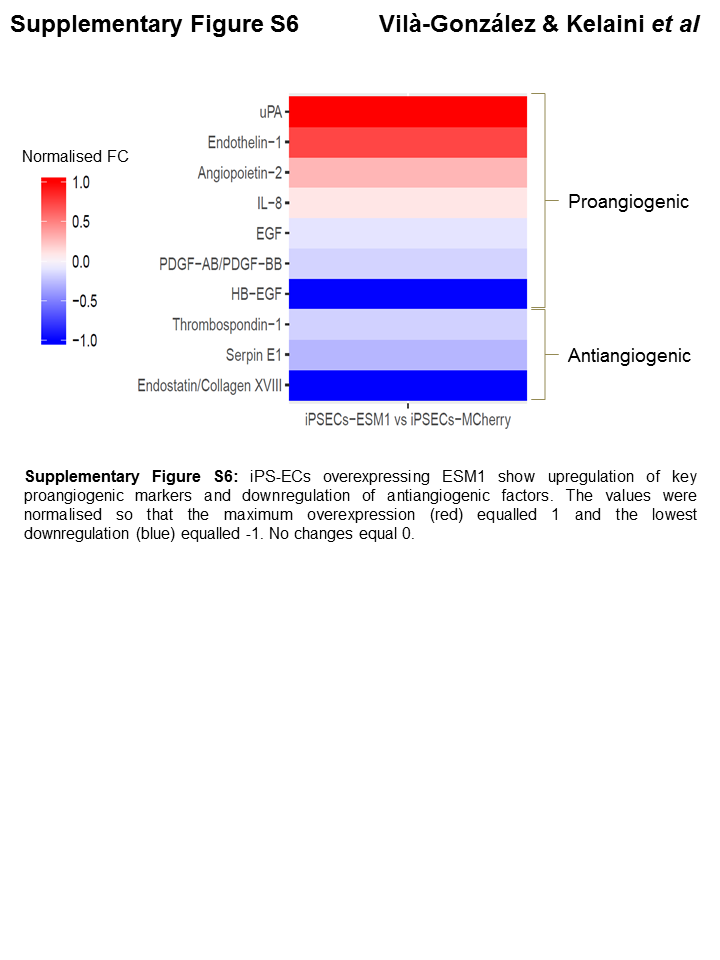

Supplement: Supplementary file 7 — Supplementary Figure S6: iPS‐ECs overexpressing ESM1 show upregulation of key proangiogenic markers and downregulation of antiangiogenic factors. The values were normalized so that the maximum overexpression (red) equalled 1 and the lowest downregulation (blue) equalled −1. No changes equal 0. [file STEM-37-226-s007.TIF]

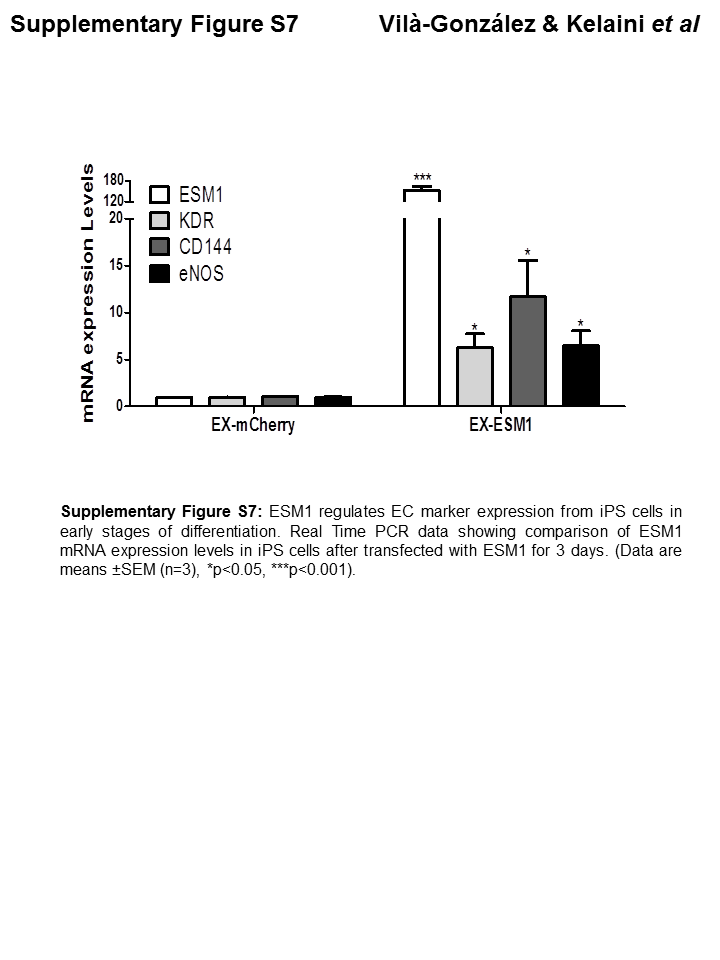

Supplement: Supplementary file 8 — Supplementary Figure S7: ESM1 regulates EC marker expression from iPS cells in early stages of differentiation. Real Time PCR data showing comparison of ESM1 mRNA expression levels in iPS cells after transfected with ESM1 for 3 days. (Data are means ±SEM [n = 3], *p < .05, ***p < .001). [file STEM-37-226-s008.TIF]

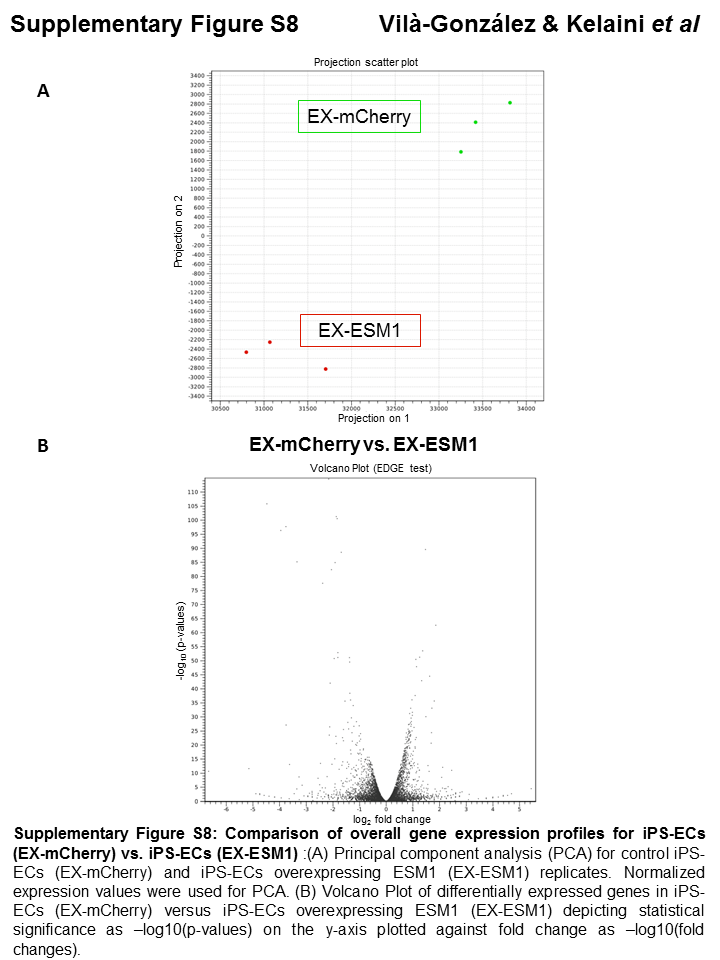

Supplement: Supplementary file 9 — Supplementary Figure S8: Comparison of overall gene expression profiles for iPS‐ECs (EX‐mCherry) vs. iPS‐ECs (EX‐ESM1):(A) Principal component analysis (PCA) for control iPSECs (EX‐mCherry) and iPS‐ECs overexpressing ESM1 (EX‐ESM1) replicates. Normalized expression values were used for PCA. (B) Volcano Plot of differentially expressed genes in iPSECs (EX‐mCherry) versus iPS‐ECs overexpressing ESM1 (EX‐ESM1) depicting statistical significance as —log10(p‐values) on the y‐axis plotted against fold change as —log10(fold changes). [file STEM-37-226-s009.TIF]

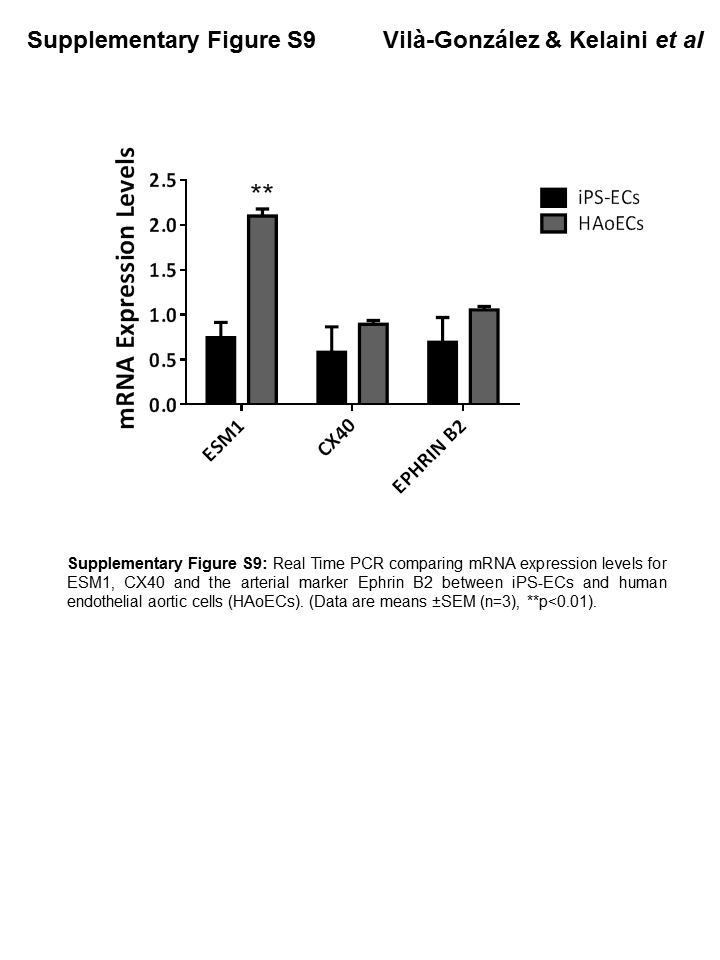

Supplement: Supplementary file 10 — Supplementary Figure S9: Real Time PCR comparing mRNA expression levels for ESM1, CX40 and the arterial marker Ephrin B2 between iPS‐ECs and human endothelial aortic cells (HAoECs). (Data are means ±SEM [n = 3], **p < .01). [file STEM-37-226-s010.TIF]

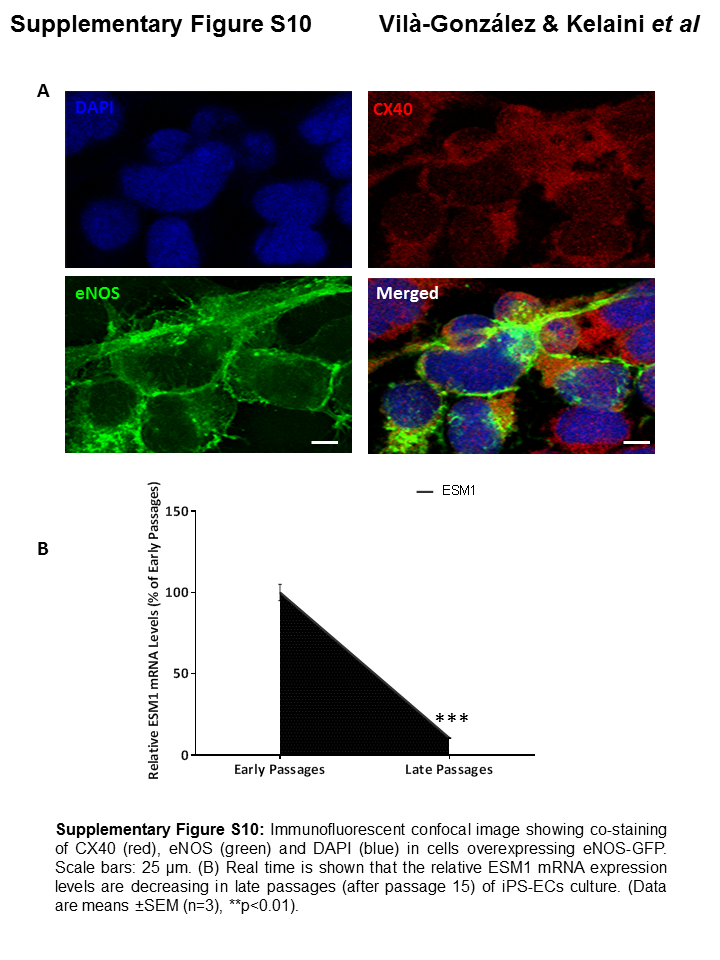

Supplement: Supplementary file 11 — Supplementary Figure S10: Immunofluorescent confocal image showing co‐staining of CX40 (red), eNOS (green) and DAPI (blue) in cells overexpressing eNOS‐GFP. Scale bars: 25 μm. (B) Real time is shown that the relative ESM1 mRNA expression levels are decreasing in late passages (after passage 15) of iPS‐ECs culture. (Data are means ±SEM [n = 3], **p < .01). [file STEM-37-226-s011.TIF]
